# Tunable Plug‐and‐Play Meta‐Nanogenerator Materials for Multi‐Range Force Measurements

**DOI:** 10.1002/advs.202600009

**Published:** 2026-01-20

**Authors:** Roshira Premadasa, Pouya Almasi, Samriddhi Ghimire, Wenkui Dong, Chenjie Zhang, Pengcheng Jiao, Qianyun Zhang

**Affiliations:** ^1^ Department of Civil Engineering New Mexico State University Las Cruces NM USA; ^2^ School of Civil and Environmental Engineering Nanyang Technological University Singapore; ^3^ State Key Laboratory of Ocean Sensing & Ocean College Zhejiang University Zhoushan Zhejiang China

**Keywords:** force sensing, mechanical metamaterials, multifunctional materials, triboelectric nanogenerators

## Abstract

Accurate assessments of mechanical forces are crucial for the design, operation and maintenance of long‐lasting engineering systems. Conventional force sensors that are deployed in engineering applications suffer from multiple shortcomings including high power consumption, poor integrability, costly, limited force sensing ranges and lack multifunctionality. In this study, we present a fully integrated, tunable meta‐nanogenerator‐based sensory system in a plug‐and‐play manner for multi‐range force measurement. At the core of this system is a novel multifunctional meta‐triboelectric material capable of quantitative and multi‐range force detection. By integrating a triboelectric nanogenerator into the tunable mechanical metamaterial design, the proposed system actively performs self‐powered force sensing while serving as a load bearing component. By effectively tuning the geometrical parameters of the proposed system, the force sensing range can be tuned to perform application‐specific sensing. A modular design is employed for the proposed system, where the 3D printed mechanical and electrical components serve as individual parts that can be independently fabricated and replaced, thus enhancing integrability and system integration. Through theoretical models, numerical simulations and experiments, the mechanical and electrical performance of the proposed system is demonstrated for different operating deformations and frequencies. Artificial intelligence models are utilized to analyze the electrical signals and to accurately determine the forces solely using the voltage signals generated. The versatility of the proposed system is demonstrated through applications in civil engineering such as bearing pads, in mechanical engineering such as shock absorbers, and as neuromuscular rehabilitation equipment in biomedical engineering. Furthermore, the performance of hierarchical arrays of the proposed system is demonstrated for enhanced and simultaneous multi‐range force sensing. The proposed system offers a paradigm shift in force sensing for intelligent and smart engineering systems with self‐powered and multifunctional capabilities.

## Graphical abstract

The multifunctional and tunable meta‐nanogenerator material system combines a mechanical metamaterial and a triboelectric nanogenerator enabling self‐powered, real‐time force sensing across application‐specific ranges. Geometrical tuning adjusts stiffness and the force sensing range, while modular integration streamlines assembly. The generated electrical signals support AI‐driven force predictions, enabling deployable, wide‐range force monitoring.

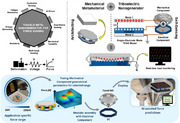

## 1 Introduction

Precise determination of mechanical forces that are being induced on engineering systems in the fields of civil, mechanical, aerospace, biomedical engineering [1, 2] etc. is of primary importance when it comes to the aspects of design, operation and maintenance. Force quantifying sensors play a pivotal role in determining the deformation, failure and adaptation behavior of engineering systems. For example, in the field of civil engineering, force measurements are pivotal in pre‐determining overloading of infrastructure during structural health monitoring. In addition to this, concentration of stresses and the fatigue performance of structures are monitored utilizing force sensors [3, 4]. During the design phase of certain structural elements, these sensors are used to do performance testing of structural elements prior to erection. The infrastructure space varies all the way from buildings, to bridges, tunnels and further. In mechanical and aerospace engineering systems, in addition to the utilization of these sensors in conventional systems for load capacity testing and health monitoring, the application space extends to multiple applications including tactile feedback in robotics, flight stress evaluation in aerospace structures, and to load control and safety in precision manufacturing. The conventional force sensors that have been deployed in engineering for decades utilize piezoelectric elements [5, 6, 7], capacitor technologies [8, 9, 10], inductive methods [11, 12, 13] etc. Even though these conventional sensors are known for their reliability and sensitivity, they possess multiple shortcomings including the limited flexibility in material selection [14, 15, 16] (For example, piezoelectric sensors are limited to specific materials like lead zirconate titanate—PZT), requirement of external power sources [17, 18], expensive and difficulties in integration with mechanical components [19] etc. One of the key drawbacks in these sensors is that they have a limited force sensing range in the low frequency domain, which stands as a limitation to use in high force applications such as seismic activities and certain machinery operating in low frequencies. In fact, since the existing sensors are limited to a fixed force sensing range, unexpected high impact loads tend to damage the sensor. Furthermore, the associated bulky electronics not only consume a lot of power but are also not resistant to harsh environmental conditions. These drawbacks highlight the need for a new class of multifunctional force sensors that are self‐powered, mechanically sound, easily integrable into the engineering system, and operating in the low‐frequency domain. In this context, multifunctional materials that are directly integrated into the engineering structure, specifically tailored to possess sufficient mechanical performance to bear loads while performing low‐frequency self‐sensing activities creates a paradigm shift in force sensing.

Mechanical Metamaterials (MMs) emerge to be promising in this context. MMs are architected materials that are geometrically tailored, with periodic arrangements of unit‐cells that outperform the intrinsic mechanical behavior of conventional materials [20]. The flexibility in the tunability of the shape enables robust [21, 22, 23, 24] and scalable [25, 26, 27, 28] designs with exceptional mechanical performance, thus resolving issues related to resilience in extreme conditions and integrability. The capability of MMs to achieve high strength‐to‐weight ratios [21, 29, 30, 31] with optimized material usage [32, 33, 34, 35] further justifies the need to synergize MMs in engineering applications to achieve low‐cost designs. Furthermore, the design freedom in material selection and geometry enabled by MMs allows the fabrication of materials that not only possess structural integrity, but also actuation and self‐sensing capabilities. Due to these reasons, MMs are well known to be used in developing multifunctional materials for sensing applications [36, 37, 38, 39, 40, 41]. In sensing applications, triboelectric nanogenerators (TENGs) have been proven to be a very viable option for sensing in a wide range of engineering applications such as civil engineering [42, 43, 44], mechanical and aerospace [45, 46, 47], biomedical engineering [48, 49, 50] etc. In TENGs, mechanical energy is directly converted to electrical energy utilizing the inherent triboelectric properties of materials [51], thus standing out among other conventional methods used in the context of sensing. TENGs operate by combining triboelectrification and electrostatic induction together. Triboelectrification occurs when tribo‐positive and tribo‐negative material come into contact with each other. At the time of contact, these two materials are oppositely charged. When the two materials are separated, charge transfer occurs by the electrostatic induction effect, leading to the development of an alternating electrical current [52]. The developed electrical signals are used for energy‐harvesting and sensing. The availability of a large range of material choices in the triboelectric series [53, 54], ranging from metals to polymers and further, enables the TENG to be a very versatile approach for sensing in the engineering domain. Furthermore, since engineering systems and structures have different deformations or movements, the multiple modes that the TENG [55] can operate in further justifies its compatibility in engineering applications. However, in order to actually use TENGs in engineering applications, they have to be structurally compatible with the system.

By utilizing the concepts of MMs, TENGs can be architected to be structurally sounds while also being designed to achieve specific displacements to tailored loads to create contact between the tribo‐pair, thus creating the platform for TENGs to operate and generate signals under mechanical excitations. This synergy between MMs and TENGs opens an avenue for multifunctional materials with load bearing and real‐time sensing capabilities, eliminating the requirement for external transducers in conventional force sensing systems. These multifunctional materials are named as meta‐tribo materials [56], where the MM itself inherits an intrinsic TENG with the key materials of the MM itself acting as dielectrics for triboelectrification. Although there is actual potential for meta‐tribo materials for force sensing applications, the existing literature in this space is mainly focused on either energy harvesting [56, 57, 58, 59, 60] or performing sensing by obtaining trends in the electrical outputs for qualitative judgements, rather than getting actual quantitative measurements of the forces induced [24, 61, 62, 63]. The electrical signals demonstrated for sensing tasks by previous work are solely to obtain qualitative judgement by the signal peaks that arise during unexpected loading conditions. Appropriate quantitative readings are necessary to determine the root of the cause and potential damage to the engineering structure. Furthermore, the proposed designs in previous work are monolithic material systems with no flexibility to replace components within them; a crucial aspect to be addressed in multifunctional material systems when implemented in real‐world applications. A modular assembly with decoupled mechanical and electrical components that serve as a unified material system to achieve multifunctionality is necessary in such systems. Furthermore, given the wide design space available in MMs, previous studies have not attempted to leverage the tunable space available to perform multi‐range sensing, a crucial aspect even conventional force sensors can't perform without additional sensors. Due to the highly non‐linear nature of the mechano‐electrical behavior in meta‐tribo materials, previous work has not reported relationships between the mechanical forces and electrical signals, thus limiting their applicability in the engineering domain.

To overcome the above‐mentioned challenges in conventional force sensors and previous meta‐tribo work, we introduce a fully integrated, tunable meta‐nanogenerator‐based sensory system in a plug‐and‐play manner for multi‐range force measurement that brings the vision of multifunctional force sensors into reality. This class of multifunctional sensory system can serve as mechanical/structural components in engineering systems while simultaneously determining mechanical loads, offering a robust, lightweight and self‐sensing alternative to conventional force sensing systems. Additionally, leveraging the design freedom in MMs, the system can be geometrically tuned for tailored force ranges, displacements and operational frequencies, thus making them capable of being utilized in a wide domain of engineering applications. Furthermore, multi‐range force sensing is demonstrated in this study, where the proposed system can sense in multiple tailored force ranges simultaneously, eliminating the need for multiple sensors in the system as done by conventional methods. The multi‐range sensing capability is achieved by seamlessly arranging hierarchical arrays of the sensors, tuned to sense different force ranges. In addition to all these added features, rather than designing the sensor as a single component, a modular approach is utilized in the design which is inspired by plug‐and‐play systems [64]. The proposed system is designed to address integrability limitations of force sensors in engineering applications by implementing a modular approach to the material system where the mechanical and electrical components can be completely decoupled and replaced. The proposed modular system facilitates the replacement of individual components in the sensor for upgrading or maintenance. Furthermore, system integration is done by employing an AI model to the mechano‐electrical behavior to predict the force values from the electrical signals. The transparent AI model employed provides closed‐form equations which can be easily deployed for effectively determining forces in engineering applications in real‐time.

**FIGURE 1:**
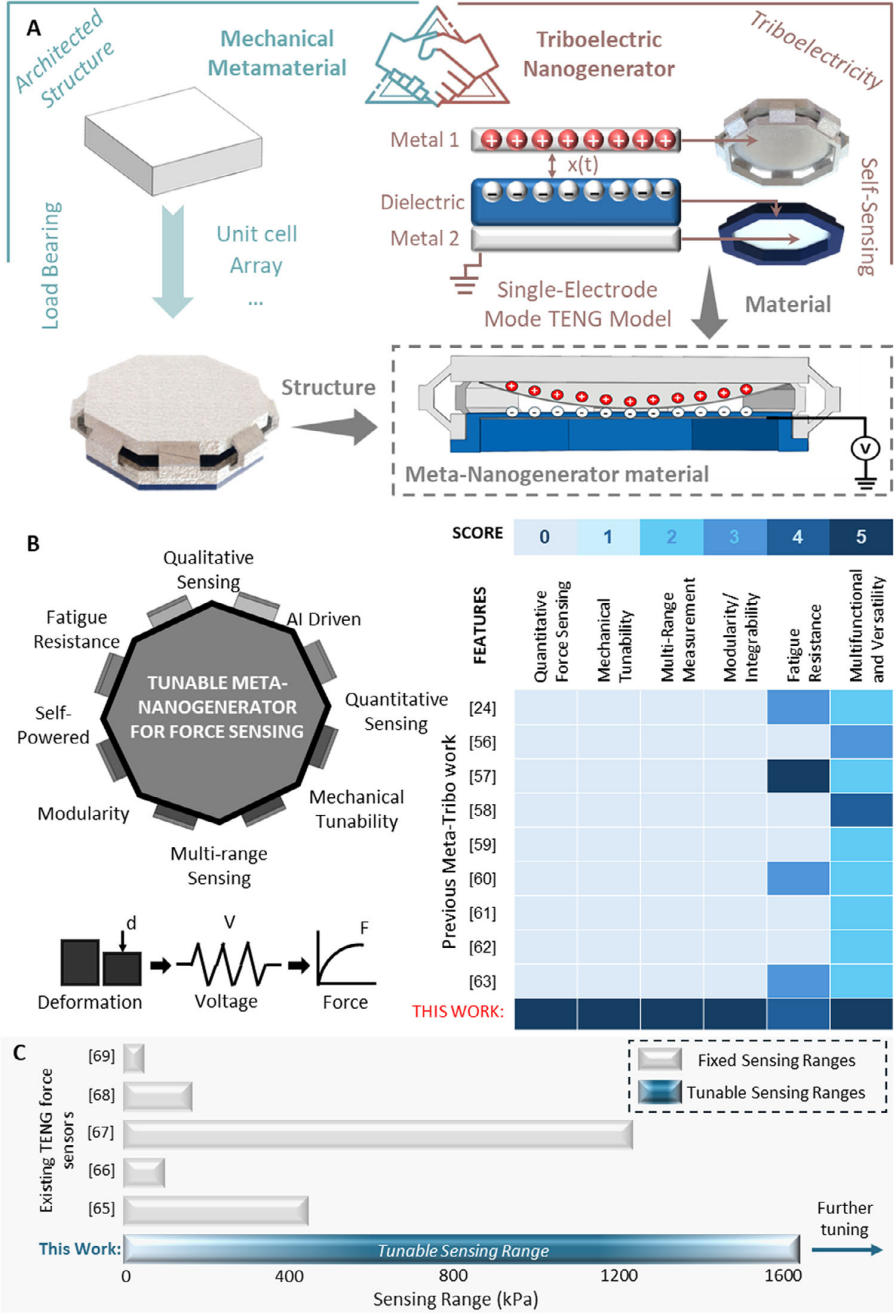
**Concept of the proposed system**. a) Meta‐Nanogenerator materials for force sensing. Significance of this work: b) Features of the proposed system and semi‐quantitative comparison with existing meta‐tribo work c) Comparison of force sensing range (kPa) with existing TENG force sensors.

Figure 1 displays a graphical representation of the vision of this study. As shown in Figure 1a, the material used for the specific engineering application is architected into the shape that possesses the required mechanical properties and equipped with the secondary component of the sensor which is made of the material that possesses the required electrical properties to perform the sensing task. The proposed system functioning as a single multifunctional system, can function as a structural component that self‐monitors the forces induced on it by converting the mechanical energy during deformation into electrical energy, outputting a signal that can be directly correlated to the force. As demonstrated in Figure 1b, in comparison to existing literature on meta‐tribo material‐based technologies, the proposed system covers a wide range of functionalities and properties that a multifunctional system requires. The features were evaluated based on a semi‐quantitative scoring method from 0–5 with 5 being the highest score in terms of performance. The evaluation criteria for the scores for each feature are elaborated in Table S1. Figure 1c demonstrates a comparison of the force sensing ranges with existing triboelectric force sensors. The tunability of the proposed system overcomes the limitation of existing triboelectric sensors having fixed force sensing ranges [65, 66, 67, 68, 69] while also surpassing the highest reported force sensing range [67] by a TENG force sensor. An in‐depth comparison of this work against existing triboelectric force sensors and meta‐tribo work is given in Table S2. The table further displays the advantages of meta‐tribo materials against high performing triboelectric force sensors. While conventional triboelectric force sensors excel in terms of sensing range and sensitivity compared to most meta‐tribo materials, these gains are achieved at the expense of tunability, multi‐functionality and multi‐range sensing capabilities. These sensors achieve high sensing capabilities by relying on complex materials and fabrication methods, which will have cost implications and limit scalability for deployment on a large scale. Most importantly, the employed TENG modes such as contact‐separation mode or sliding mode that require electrical connections with multiple layers within the TENG system, refrain the sensors from being modular and easily integrable in actual applications. On the other hand, the comparison in Table S2 clearly demonstrates that meta‐tribo materials achieve multiple capabilities within a single platform by utilizing a MM with inherent triboelectric layers. However so far, no single triboelectric or meta‐tribo material has been able to achieve multifunctionality with accurate force sensing capabilities, modularity, tunability and multi‐range sensing capability within a single platform. The summary clearly highlights this gap while underscoring the significance of this work.

## 2 Results and Discussions

### 2.1 Mechanical Component Design and Performance

**FIGURE 2:**
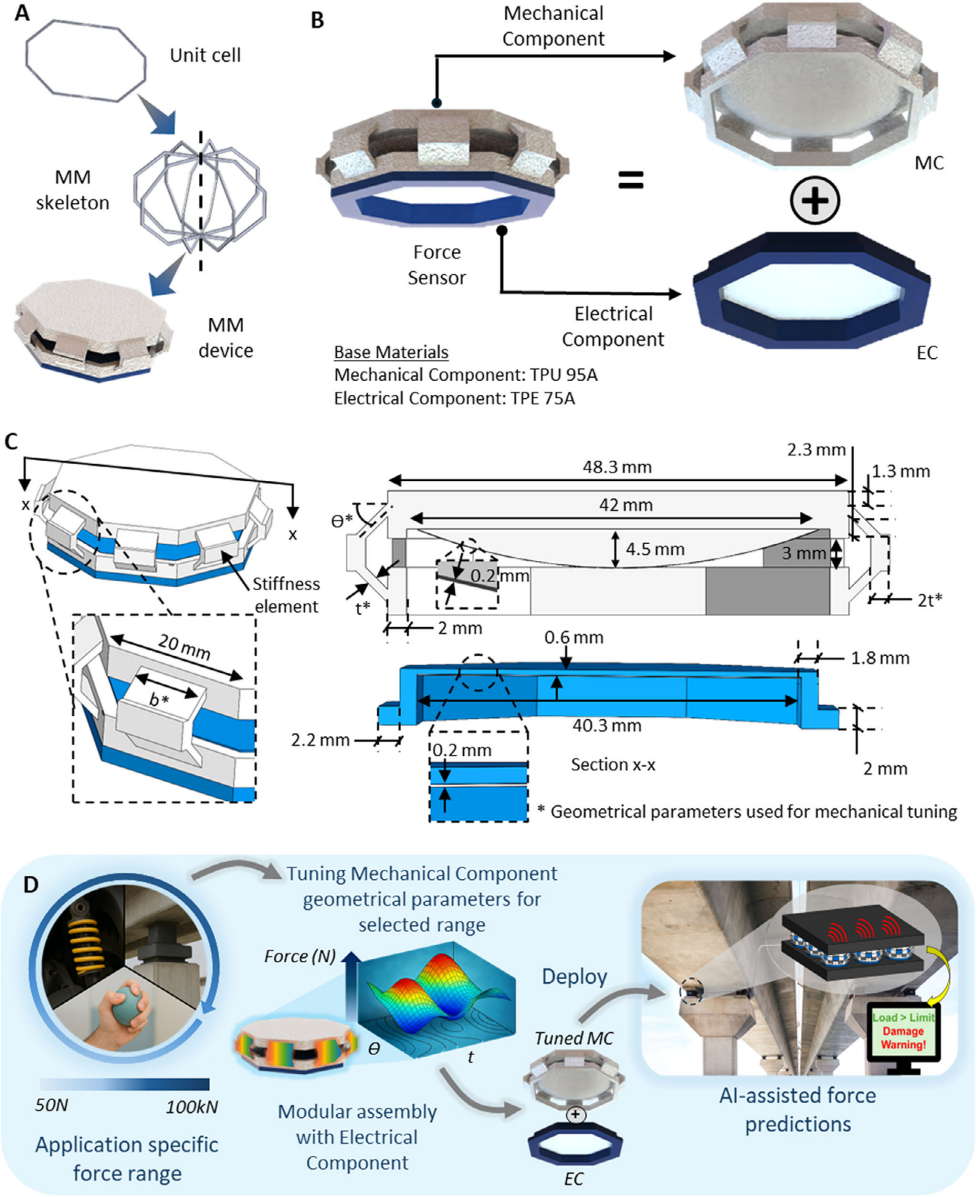
Design of the proposed system a) Unit‐cell design b) Components c) Geometrical parameters d) Conceptual workflow of the proposed system.

Honeycomb MMs [70, 71] have a proven track record of having excellent mechanical properties. The MM design used in this study is a convex honeycomb in a volumetric shape as shown in Figure 2a and comprises of 4‐unit cells that are connected at one central axis. These shapes are popular in the MM domain for having excellent specific strength and stiffness [72]. And in addition to that, these shapes deliver exceptional dynamic mechanical performance such as energy absorption [71] and impact resistance. At the same time, the honeycomb structures inherent good compressibility due to the nature of their cellular design [73]. This exceptional mechanical behavior and tunability makes the convex honeycomb structure an ideal candidate for engineering applications. As shown in Figure 2b, the proposed system consists of composite multifunctional material equipped with two components, the mechanical and electrical components. Rather than fabricating the proposed system as a single component, it utilizes a modular design, where individual components are assembled to create the final unit. The design is inspired by plug‐and‐play systems, where the components can be removed and replaced. This facilitates integrability, repairs and maintenance. The base material for the mechanical component (MC) explored in this study is the polymer, thermoplastic polyurethane with shore hardness of 95A (TPU 95A) [E = 61.88 MPa]. However, MC can be fabricated using different materials and be effectively used based on the requirements of the application (Refer Section S2.2). The base material of the electrical component (EC) is made of highly flexible material; thermoplastic elastomer with a shore hardness of 75A (TPE 75A) [E = 37.62 MPa]. The EC is specifically made of this soft material to avoid any interference from the EC on the mechanical performance. The size of the proposed system is scalable based on the requirements of the application it is being used for, and for demonstration in this study, the dimensions used are as displayed in Figure 2c. The key component of the MC responsible for the stiffness of the proposed system is the arm like element marked in Figure 2c., hereafter mentioned as the ‘stiffness element’. The performance of the MC is directly dependent on the geometrical parameters of the stiffness element.

All geometrical parameters of the MC shown in Figure 2c. are kept constant to allow complete mechanical performance tunability to be done using only the width‐b*, thickness‐t* and the angle‐Ө* of the stiffness element. It is worth mentioning that other geometrical parameters such as the number of stiffness elements (8 in this study) can be varied to achieve further tunability of the MC. The MC is designed with an elliptical dome (coated with silver) with a height of 4.5 mm and a diameter of 42 mm. The dome design is mainly influenced by the requirements in the electrical design to have a varying contact surface area with the EC at different deformations. The flat area of the EC is 0.6 mm in thickness and is specifically thin to avoid any significant effect from the EC to the forces induced, when the dome and the EC come into contact during deformation (Refer to Section S2.2 for the behavior of the proposed system with and without the EC).

The theoretical equations for the force vs displacement behavior of the MC are derived as follows (Refer to Section S2.1 for the detailed derivation):

(1) $$F=\left(\frac{2bEt^{3}}{{\left(\frac{2.3}{Sin\left(\theta \right)}+t\right)}^{3}\cos\left(\theta \right)}\right)\;x\;\Delta N$$

here;

E = Modulus of Elasticity of the material

B = width of the arm

T = thickness of the arm

Ө – angle of the arm

Δ = vertical deformation at the free end

In this study, the maximum deformation of the MC is limited to 3 mm.

Therefore, the maximum force (F_max_) on the MC is;

(2) $$F_{max}=\left(\frac{6bEt^{3}}{{\left(\frac{2.3}{Sin\left(\theta \right)}+t\right)}^{3}\cos\left(\theta \right)}\right)\;N$$

As shown in Equation (2), the maximum force is completely tunable by the material property E, and the geometrical parameters b, t and θ. This mechanical tunability empowers the design of application‐specific material systems with control over load bearing capacity and deformation behavior. Figure 2d demonstrates the conceptual workflow of the proposed system. Initially the application‐specific operational force range is identified. The MC component is tuned using geometrical parameters to achieve the desired force range. The tuned MC now serves as a load bearing component for the specific application. The tuned MC is equipped with the universal modular EC component in a plug‐and‐play manner to enable precise self‐powered force sensing capabilities. Once deployed, the proposed system enables AI‐assisted force and performance monitoring, serving as a multifunctional element.

**FIGURE 3:**
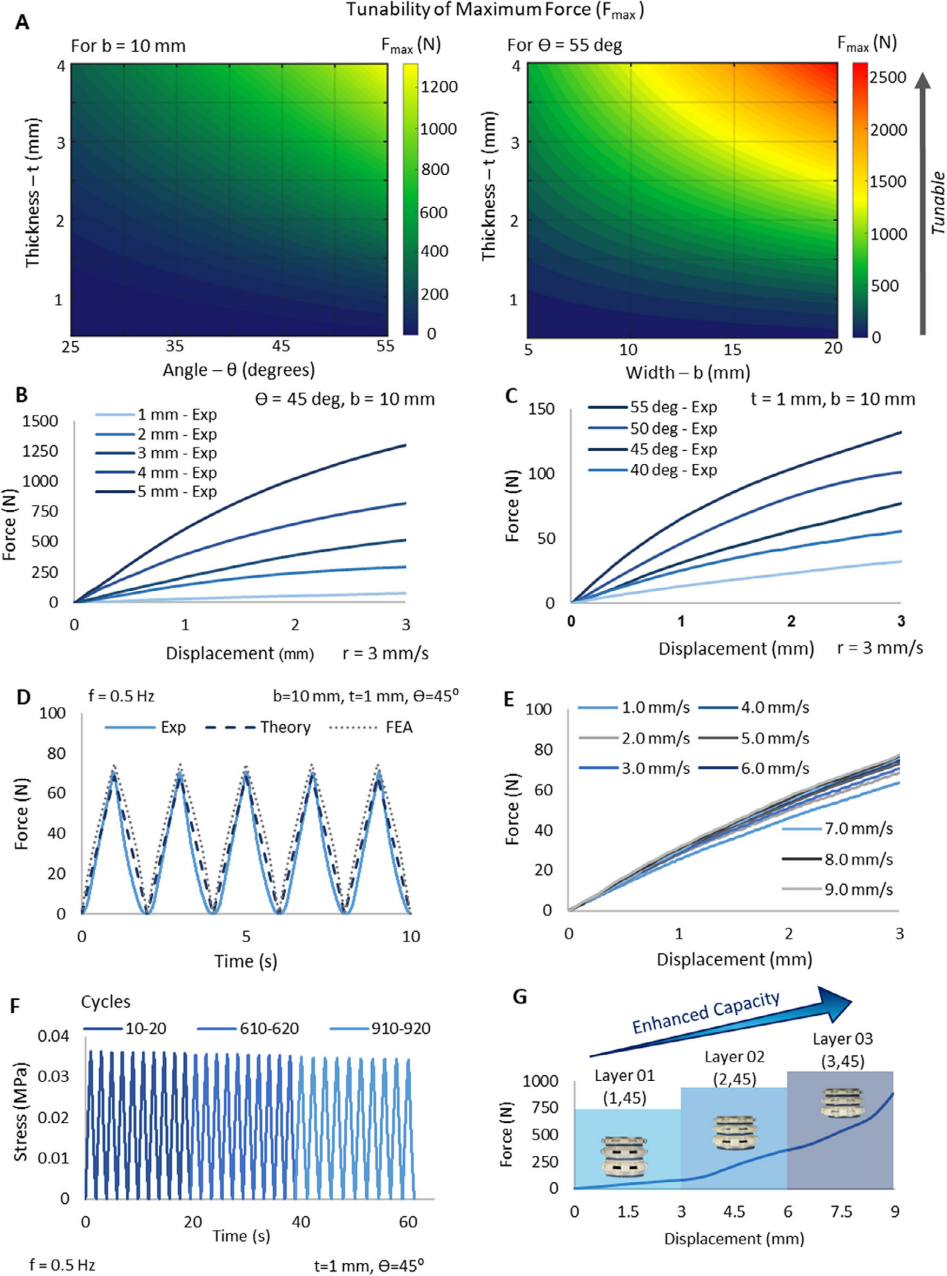
Mechanical performance of the proposed system a) Contour plots demonstrating mechanical tunability of maximum force with geometrical parameters t, b and Ө b) by varying angle c) by varying thickness d) under cyclic loading e) by varying displacement rate f) fatigue performance g) Hierarchical arrays for enhanced force and deformation behaviors.

Given in Figure 3a are the design contour plots to facilitate the determination of the geometrical parameters for tailored maximum forces. The contour plots were obtained from the derived Equation (2). As it can be seen from the contour plots, the maximum force increases when b, t and Ө increases. Hereafter, the designs of the proposed system are symbolized as (t, Ө), which represents the combinations of t and Ө used in the specific design. Figure 3b shows the experimental results of the force‐displacement behavior of the units when the t is varied while keeping Ө constant at 45^0^. As it can be seen, the maximum force increases with increasing t. A similar behavior is observed by varying Ө while maintaining t constant at 1 mm as shown in Figure 3c. As shown in Figure S2a, the geometry can be tuned to achieve a maximum force of 3297 N (1707 kPa) and is further tunable. The plots for the maximum forces along with error bars are given in Figure S2e, f to demonstrate the repeatability of the measurements. In order to demonstrate the validity of the theoretical and experimental results, Finite Element Analysis (FEA) was conducted. The FEA results plotted against the experimental and theoretical results for a (1,45) sample tested in a cyclic test at 3 mm deformation is shown in Figure 3d. As shown, the experimental, theoretical and FEA results are in good agreement with each other with theoretical and FEA results having a difference of ‐2.98% and 3.64% with the experimental results respectively at the peak. In order to demonstrate the effect of the displacement rate on the behavior of force‐displacement relationship of the MC, tests were conducted on the MC for different displacement rates varying from 1 mm/s to 9 mm/s. This range of displacement rates was selected to accommodate the selected operating frequency range of the proposed system from 0.5 Hz to 1.5 Hz. Figure 3e shows the experimental results obtained for the different loading scenarios. It was observed that the forces induced on the MC was increasing proportional to the displacement rate, with a percentage increase of 17.58% in the maximum force between the samples tested at 1 mm/s and 9 mm/s, indicating that the displacement rate has a significant effect on the behavior of the MC and that it is crucial to take this effect into consideration when using the sensor to determine forces. Low cycle fatigue testing for a total of 1000 cycles was performed according to ASTM D7791 to evaluate the fatigue performance of the sensor. The fatigue test results are illustrated in Figure 3f. It was observed that the MC exhibits good fatigue endurance after 610 and 910 cycles respectively. The proposed system also demonstrated excellent energy absorption capabilities. As shown in Figure S2d, maximum energy absorption densities for the (1,45), (2,45) and (3,45) units are 22 kJ/m^3^, 94 kJ/m^3^, and 454 kJ/m^3^ respectively according to experimental data, indicating the tunability and effective energy absorption capabilities of the proposed system.

Not limited to single units, multiple units can be arranged in arrays to create hierarchical material systems with unique and enhanced force and deformation ranges. By unique arrays, the material system can be tuned to achieve tailored force‐displacement behaviors with targeted load bearing capacities. As shown in Figure 3g, an array of the proposed system was tuned to achieve a target enhanced force of approximately 900 N at a deformation of 9 mm. This unique behavior was achieved by a 3‐layer system with (1,45), (2,45), and (3,45) units arranged in order from top to bottom.

### 2.2 Electrical Component Design and Performance

The EC design of the proposed system was designed to enable the triboelectric effect during mechanical excitations. The TENG works in a mechanism where the surfaces of two materials get oppositely charged when in contact with each other and generates a voltage, thus creating an electrical signal that can be used for sensing. The ability of the materials to create these distinct charges depends on the materials’ electron affinity and appropriate selection of materials from the triboelectric series.

As shown in Figure 4a, similar to a typical single electrode model, the proposed system also has a dielectric‐to‐dielectric single electrode mode TENG. By utilizing the single electrode mode, the MC and EC are fully decoupled from any physical electrical connection between each other to facilitate modularity.

FIGURE 4Electrical performance of the proposed system a) TENG design b) FEA using COMSOL Multiphysics c) Experimental testing setup and voltage generation under cyclic loading d) Voltage signal generation at different displacements e) Voltage output and corresponding contact area at different deformation amplitudes f) Voltage signal generation at different frequencies. g) Variations in the generated voltage values as temperature increases from 22.4 °C to 40°C h) Plots showing the correlation between the sensor voltage and temperature variations i) Fatigue performance. Force Vs Voltage behavior for t = 1,2,3 mm designs at j) 0.5 Hz k) 1.0 Hz l) 1.5 Hz.

**Figure:**
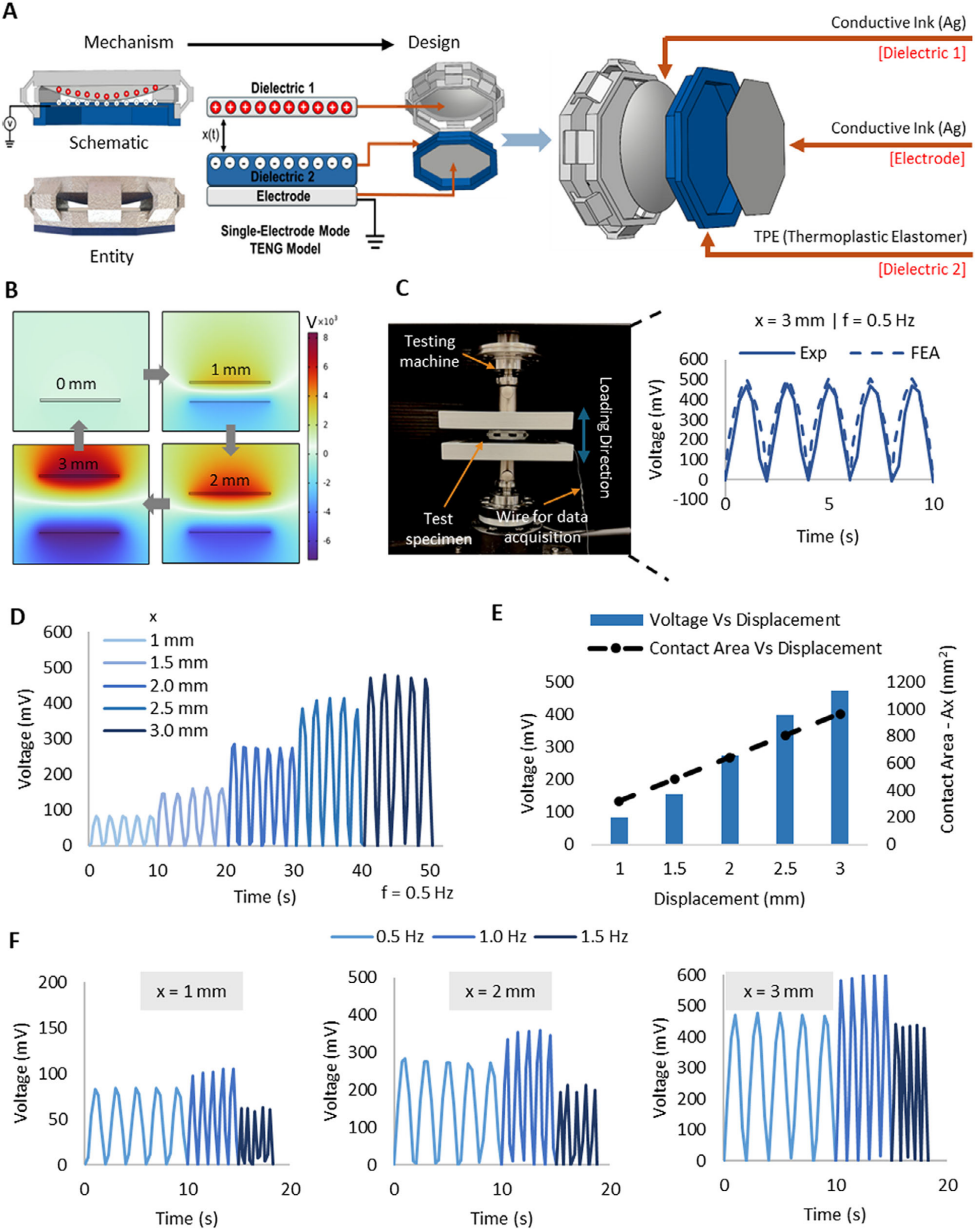

**Figure:**
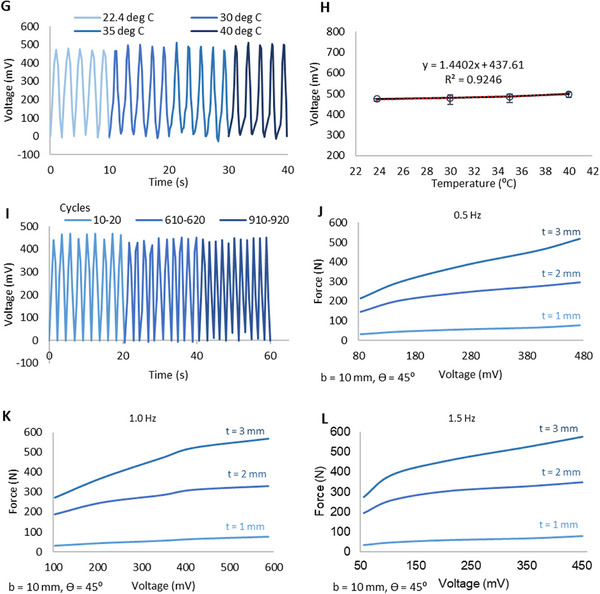

The theoretical behavior of the TENG in the proposed system is explained below for the dielectric to dielectric model demonstrated in Niu et al., (2014) [74]. Here the dielectric 1 is the MC and a thin layer of silver (0.2 mm) was coated on the dome of the MC to achieve tribo‐positivity. The base material of the EC (TPE) is itself a tribo‐negative material and behaves as the dielectric 2. A thin silver layer (0.2 mm) is available at the bottom of the EC to create the primary electrode to ground the charges generated. This primary electrode is connected to a reference electrode (in this case, the reference electrode is the ground) via an external load (device measuring the signals). The primary motion is in the dielectric 1 (MC) moving in the vertical direction due to the mechanical forces, while the dielectric 2 and the two electrodes are stationary. This motion forms the varying distance x(t) between the tribo‐materials (with the maximum x being 3 mm in the proposed system). When the two dielectrics get into contact with each other, contact electrification in the tribo‐pair with a net charge of +σ and ‐σ forms at the surfaces of the dielectric 1 and 2 respectively. Here, it is assumed that charges are well distributed on the surfaces of the materials with negligible decay, owing to the excellent insulation properties of the materials and the nature of contact electrification [75, 76]. At the instance of contact, the charges with opposite signs on the surface coincide with each other approximately on the same plane, since the charges are confined only to the surfaces at this point. At this instance, there is practically no potential difference between the two dielectrics. When the two surfaces of the dielectrics separate (the gap that is created between dome and the EC when the load is released), a potential drop is created. This potential difference occurs due to the electrostatic induction effect when the two materials separate from each other. Due to this potential difference, free electrons flow from the electrode of the EC to the ground. The free electrons flow back when the potential drop generated by the triboelectric charges disappears as the gap closes. This back and forth flow of electrons generates an AC output which is directly used for sensing [52] by the proposed system as shown in Video S1. FEA done to demonstrate behavior of the proposed system is shown in Figure 4b. As it can be seen in the figure, when there is no separation between the MC and EC (x = 0 mm), there is no electric potential developed. As the two materials separate from each other, the electric potential increases until it reaches its maximum separation distance (x = 3 mm). The cycle goes on with the materials contacting and de‐contacting each other. Further details on the FEA is elaborated in Section S4.2.

Here, Q can be referred to as the charges (Q = σA_x_ where σ: surface charge density and A_x_: contact surface area) that are transferred between the primary and reference electrodes, the potential difference that drives the electron transfer to happen can be referred to as V. Due to the capacitance (C) formed between the primary and reference electrodes, a linear relationship between the V, Q and x can be shown. The reciprocal of the gradient of the V‐Q‐x relationship is the capacitance‐C and the non‐zero intercept is the Voc (open circuit voltage) value. Therefore, as shown in [74], V‐Q‐x relationship for the single electrode mode TENG can be expressed as below:

(3) $$V=-\frac{1}{C}\;x\;Q+V_{OC}$$

(4) Where,Q=σAx

Since V = 0 at the short circuit condition, the V‐Q‐x relationship is

$$0=-\frac{1}{C}\;x\;Q_{SC}+V_{OC}$$

(5) $$Q_{SC}=V_{OC}\;x\;C$$

where Q_SC_ is the short circuit transferred charge.

The capacitance C is nearly a constant(C_0_) since the electrodes are fixed.

By combining Ohm's law with the V‐Q‐x relationship of the single‐electrode TENG, a relationship can be derived to take the load properties into consideration.

(6) $$V=IR=R\frac{dQ}{dt}$$

By combining Equations (3) and (6), we get:

(7) $$R\;\frac{dQ}{dt}=-\frac{1}{C}\;x\;Q+V_{OC\;}\left(x\right)$$

By applying the boundary condition Q (t = 0) = 0 [considering that contact separation is occurring at t = 0 when the dielectric has been at x = 0 position for a long time], the following equation can be derived:

(8) $$Q\left(t\right)=\frac{1}{R}\;e^{-\frac{1}{RC_{0\;}}}\int_{0}^{t}V_{OC\;}\left(x\left(t\right)\right)e^{\frac{1}{RC_{0\;}}}dt$$

and hence, the current and voltage output equations can be derived as given below:

(9) $$I\;\left(t\right)=\frac{dQ}{dt}=\frac{V_{OC\;}\left(x\left(t\right)\right)}{R}\;-\;\frac{1}{R^{2}C_{0\;}}\;e^{-\frac{1}{RC_{0\;}}}\int_{0}^{t}V_{OC\;}\left(x\left(t\right)\right)e^{\frac{1}{RC_{0\;}}}dt$$

(10) $$V\;\left(t\right)=V_{OC\;}\;\left(x\left(t\right)\right)-\;\frac{1}{RC_{0\;}}\;e^{-\frac{1}{RC_{0\;}}}\int_{0}^{t}V_{OC\;}\left(x\left(t\right)\right)e^{\frac{1}{RC_{0\;}}}dt$$

Using the proposed system, unique voltage signals are necessary to identify different forces. These forces arise due to the different deformations that the sensor undergoes. In order to do this, the sensor is designed to have unique charge generations upon contact with different deformations. This is achieved since the contact surface area (A_x_) changes when the dome penetrates a distance (x) into the EC. The relationship between the deformation and contact surface area can be defined as in Equation (11) (Refer Section S3.1 for detailed derivation).

(11) $$A_{x}=102.5\pi xmm^{2}$$

As the dielectric 1 is designed as a dome shape, the contact surface area between the dome and the flat part of the EC (elastomer) varies depending on the deformation the sensor undergoes. The elastomer acts like a membrane wrapping around the dome. The more the dome penetrates downwards into the membrane, the more the membrane wraps around the dome with increased contact surface area. Therefore, assuming ideal contact conditions, the contact area evolution for contact electrification varies with the displacement x as shown in Equation (11).

Since Q ∝ A_x_ as shown in Equation (4), Q ∝ *x*.

Due to the increasing contact area, higher numbers of charges are developed on the surfaces for transfer, thus increasing the voltage signal generated at higher deformations (d↑ → A↑ → Q↑ → V**↑)**.

The proposed system was experimentally tested for a series of displacements to demonstrate the diversity of the signals generated. The experimental test results are shown in Figure 4d. All voltage values extracted were the average of the peak values of 5 cycles. It can be seen that the sensor generates different voltage signals for different deformations ranging from approximately 85 mV to 475 mV for 1 mm to 3 mm displacements respectively, clearly indicating the sensors capability to capture different loading scenarios. The plot with error bars is given in Figure S3b to demonstrate the repeatability of the system. Figure 4e shows the peak voltage outputs and corresponding contact areas at different deformation amplitudes, illustrating that triboelectric signal generation is strongly governed by displacement‐induced increases in real contact area.

For the proposed system to be reliable and versatile, it is required to capture unique signals based on different loading conditions. In addition to different deformation conditions, one of the key parameters is the frequency of motion. To demonstrate the diversity of signals generated for different loading frequencies, a series of experimental tests were conducted for deformations at different frequencies ranging from 0.5 Hz to 1.5 Hz. Some of the key test results are as shown in Figure 4f (An extended demonstration of the experimental results is shown in Figure S6). As it can be seen, the voltage signals generated display a non‐linear relationship with the frequencies. For all the test deformation scenarios, the sensor is capable of generating unique signals based on different combinations of frequency and displacement, thus making it possible to capture different scenarios for sensing. The peak voltage signals increase with the increasing frequency until a frequency of approximately 1.0–1.25 Hz and then starts decreasing. A similar trend is observed at higher frequencies as shown in Figure S3c. This behavior can be explained in relationship to the charge transfer rate. As shown in Equation (6), the charge transfer rate is proportional to the voltage. Therefore, an increase in the frequency will increase the charge transfer rate, which results in an increase in the open circuit voltage [77]. However, at higher frequencies (i.e beyond 1–1.25 Hz), regardless of the high charge transfer rate, some of the surface charges are incapable of getting transferred due to insufficient charge transfer time at high frequencies, thus resulting in an incomplete charge transfer [78]. Therefore, a decrease in the voltage signal can be observed. Further validation of this observation is given in Section S3.2 and Figure S3d. However, all in all, the sensor is capable of capturing a unique signal for all loading scenarios.

Low cycle fatigue testing was also conducted to test the stability of the electrical signals under long term conditions. The test was conducted for 1000 cycles at a frequency of 0.5 Hz for the 3 mm displacement scenario. As demonstrated in Figure 4i, the voltage signals remain reasonably stable after 1000 cycles after 610 and 910 cycle respectively. The presented results and models are valid for the tested conditions at room temperature (22.4 °C). However, due to different climatic conditions based on the region and season, the proposed system may be exposed to a range of temperatures, and these temperature fluctuations can impact the voltage signals generated. Therefore, we conducted experimental tests to evaluate the voltage signals generated at temperatures ranging from room temperature to 40°C. The tests were conducted for a displacement of 3 mm at a frequency of 0.5 Hz as shown in Figure 4g. Figure 4h demonstrates the averages of the voltage signal in 5 cycles. The voltage signals linearly increase with increasing temperatures, with the best fitted line in linear regression possessing a R^2^ ≈ 0.93. This behavior is attributed to the increase in charge mobilization at elevated temperatures within this temperature range that leads to increased voltage signals [79, 80]. The recorded voltage values increased by 1.1%. 2.3% and 5.1% at 30°C, 35°C, and 40°C respectively. These observations provide valuable insights into the effects of temperatures that need to be taken into consideration when employing the proposed system in real‐world applications. The correlations demonstrated in Figure 4h can potentially be useful in calibrating the system and optimizing the AI models in future work.

### 2.3 Mechano‐Electrical Coupling/System Integration

With the voltage signals being generated based on different forces induced on the proposed system, integration is necessary to couple the electrical output with the mechanical input, for effective determination of the force values by the force sensor. Based on the evaluations of mechanical and electrical performances, non‐linear relationships are observed for the combined effect of the displacement, displacement rate and forces and the displacement, frequency and voltage respectively. The experimental mechano‐electrical behavior of the system is shown in Figure 4j–l, where triboelectric voltage is directly correlated with the corresponding force at different deformation amplitudes and operating frequencies. In all cases, the force response increases nonlinearly with voltage, indicating that the generated electrical output is a result of deformation‐dependent contact behavior. In addition to deformation effects, the mechano‐electrical response also varies with frequency, as seen from the shifting force–voltage curves across 0.5, 1.0, and 1.5 Hz. For the same deformation level, the voltage produces different force outputs depending on the operating frequency, indicating that the electrical signal is not fixed for a given displacement but changes with the loading rate. This shows that the force–voltage relationship is dynamic, and the coupling between mechanical deformation and electrical output is influenced by frequency as well as displacement. It is noteworthy that this nonlinear and frequency‐dependent voltage–force trend persists across different geometric configurations (t = 1 mm, t = 2 mm, t = 3 mm) as well. Overall, based on the data collected, the correlation of the voltage value with the force value is a direct yet non‐linear relationship as shown in Figure S6.

In order to comprehensively capture the non‐linearity and to predict the forces based on the voltage signals extracted from the sensor, advanced artificial intelligence (AI) models were developed. For this study, evolutionary computation models that utilize explainable AI [81] to demonstrate the patterns hidden in the data through symbolic regression were utilized, instead of adopting conventional black box models such as artificial neural networks that don't unveil the relationships between the data. Specifically for this study, the Multi‐Gene Genetic Programming (MGGP) algorithm was adopted, which is a solid AI technique that runs based on evolutionary computation [82]. For a robust system integration, a proper dataset is necessary to effectively capture the force vs displacement and displacement rate, and voltage vs displacement and frequency relationships. For this purpose, a series of tests were conducted for displacements in the range of 1–3 mm and frequencies in the range of 0.5–1.5 Hz. The force‐displacement plots tested for a maximum displacement of 3 mm and displacement rates in 0.125 mm/s intervals for a range of 1 mm/s to 9 mm/s were obtained. The displacement rates were selected based on the operating frequency of 0.5–1.5 Hz of the sensor. By back calculating the corresponding frequencies from the displacement rates, a series of cyclic tests were conducted for these frequencies to obtain the voltage values for displacements at 0.5 mm intervals in the range of 1–3 mm.

**FIGURE 5:**
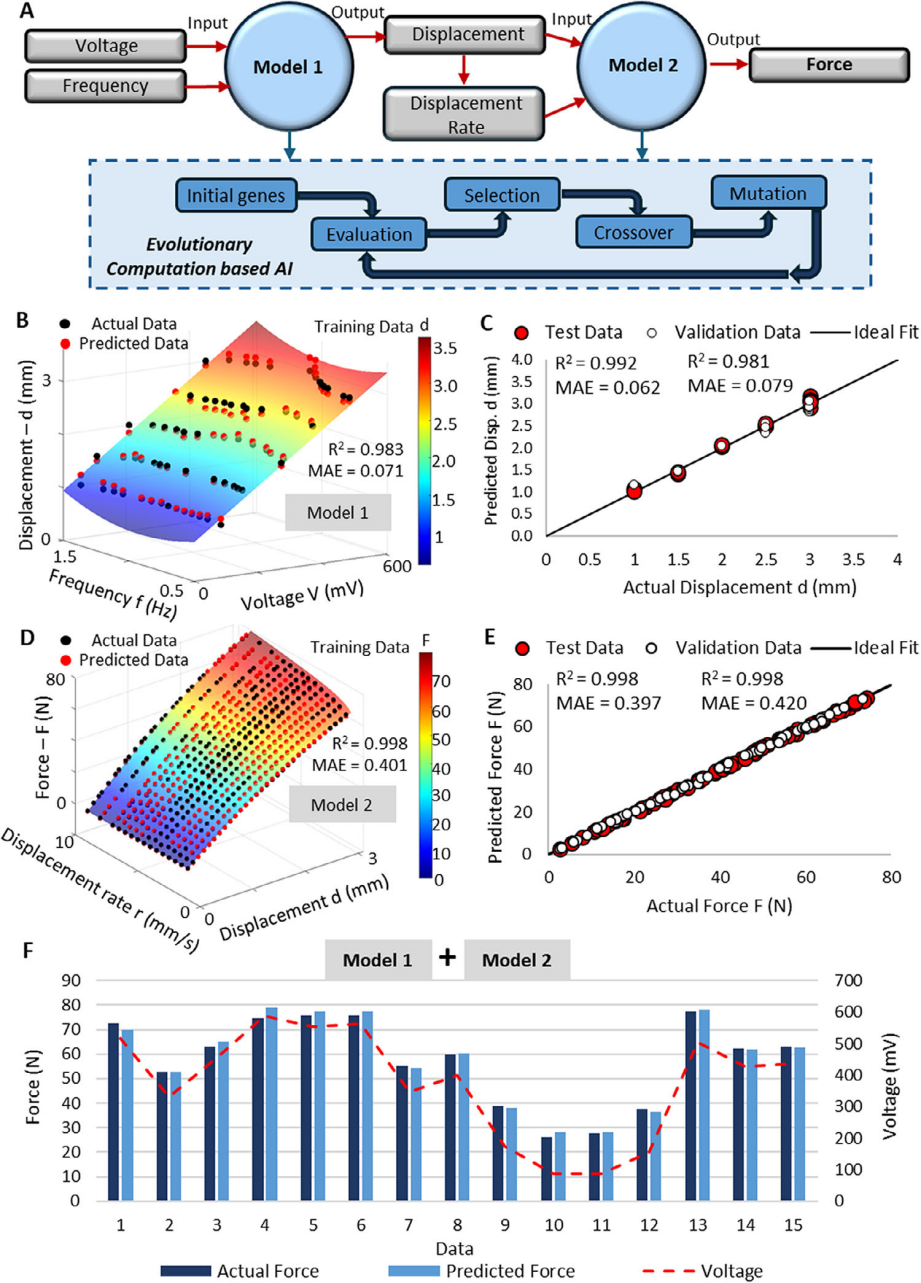
System integration using AI for force prediction a) AI evolutionary process for force predictions using the sensor signals b) Training results of Model 1 c) Validation and testing results of Model 1 d) Training results of Model 2 e) Validation and testing results of Model 2 f) Fused model for system integration tested against actual force data from a commercial load cell.

The method utilized here develops predictive equations between the target and predictor variables utilizing symbolic regression, creating a transparent platform with mathematical equations to explicitly predict the forces using the output voltage signals. As shown in Figure 5a, two AI models were trained to derive mathematical relationships between the data. Both models run based on evolutionary computation where the model assigns a set of initial genes where evaluations, selections, mutation and crossovers occur for populations over generations to generate the best fitting gene. The first AI model (Model 1) was used to predict the displacement (target variable) using the voltage and frequency as the predictor variables. The model was trained using the data specifically extracted from the cyclic tests done to evaluate the voltage signal behavior with different frequencies and displacements. The second AI model (Model 2) was trained to predict the force (target variable) using the displacement and displacement rates as the predictor variables. The dataset used was data from force‐displacement plots for different displacement rates performed experimentally. Upon training the models, the two models can be fused to develop the final prediction model. Here the predicted displacement values can be inputted into the second model along with the displacement rate calculated using the frequency to effectively determine the force predictions. For both AI models, 70% of the data was utilized for training the model, while the remaining data was split equally for validation and testing. The accuracy of the models was evaluated using two performance indexes; coefficient of determination (R^2^) and mean absolute error (MAE). Additional details on the working mechanism of the AI models and the development process are elaborated in Section S5.1.

The first AI prediction model (Model 1) trained for displacement predictions is shown in Equation (12):

(12) $$d=0.00438V\;-f\left(0.139+1.01cos\left(f\right)\right)+1.26$$

D = displacement (mm), V = voltage (mV), f = frequency (Hz)

Figure 5b demonstrates the results of the measured and predicted displacement values for the training dataset. The high prediction accuracy with negligible error explains the effectiveness of the model in accurately predicting the displacement values. The accuracy of the prediction values for the validation and testing dataset is shown in Figure 5c. With a R^2^ = 0.992 and MAE = 0.062 for the test data further validates the robustness of the prediction model.

The second AI prediction model (Model 2) trained for force predictions is shown in Equation (13). For demonstration, the (1,45) sample was selected.

(13) $$F=\frac{d}{r}\;\left(0.315r^{2}+30.9r-2.48dr\;-\;2.79\right)-0.364$$

F = force (N), d = displacement (mm), r = displacement rate (mm/s)

As shown in Figure 5d, the trained model had high accuracy with a R^2^ = 0.998 and MAE = 0.401. This indicates that the model is capable of accurately capturing the relationship between the force, displacement and displacement rate. The checks done with the unseen data (validation and testing data) (Figure 5e) also displayed a high accuracy with a MAE of approximately 0.4 N, further justifying the reliability of the model.

Now by fusing Model 1 and Model 2, a global model is created for the (1,45) design where the force values are predicted effectively by using the data from electrical signals.

(14) $$F=\mathrm{function}(d=0.00438V\;-f\left(0.139+1.01cos\left(f\right)\right)+1.26,\;r=2df)\;N$$

To test the fused model, the set of shuffled unseen data (testing data) with voltage and frequencies values only was directly input to Equation (14). The results are as shown in Figure 5f. The fused AI model was able to provide reliable prediction results using the provided testing data with an average error of only 0.44 N. This demonstrates that the proposed system, supported by the AI model, is capable of reliably determining forces across different frequencies with performance comparable to that of a commercial load cell.

Similarly, the fused model for other units can be developed as well. The models for the (2,45) and (3,45) units are shown in Section S5.1.

To verify the reliability of the closed‐form predictive models under realistic sensing conditions, an uncertainty analysis was performed by introducing varying perturbations to the model inputs as shown in Section S5.2. The results demonstrated that these perturbations produced only minimal variation in the predicted force outputs, indicating that the models are robust against typical measurement fluctuations and remain stable across the operating range. This analysis confirms that the derived models can reliably capture the underlying mechano‐electrical behavior even in the presence of input noise. To further verify that the proposed model captures the underlying mechano‐electrical behavior, a parametric study was carried out by varying both displacement and frequency within the experimental range as shown in Section S5.3. The resulting model outputs consistently reproduced the observed trends in voltage–force coupling for different operating conditions, confirming that the model effectively reflects the deformation and frequency‐dependent characteristics of the system.

### 2.4 Potential Applications Using the Proposed System

Given that the proposed system possesses mechanical tunability and accurate force sensing capabilities, it can potentially be deployed in a wide range of civil, mechanical and biomedical engineering applications for load bearing, damping and real‐time health monitoring in the low‐frequency domain. In structural engineering, structures undergo low‐frequency deformations due to pedestrian motion, wind loads or thermal expansion [83, 84]. As shown in Figure 6a, the proposed system could be used in civil engineering applications such as in bearing pads for real time monitoring of the loads induced at the supports. To demonstrate the applicability of the proposed system for such applications, lab scale experiments were conducted. As shown in Figure 6c, a bearing pad arrangement was setup with a 2 × 2 array of the proposed system embedded in between 2 bearing pad plates (150 × 150 × 10 mm) fabricated using a stiff material (PLA – Polylactic Acid). A concrete specimen (100 × 100 × 100 mm) was placed on top of the bearing pad and subjected to cyclic loading for a displacement of 2 mm at a frequency of 0.5 Hz. The signals generated from 2 diagonally arranged sensors, each from the front and back rows were monitored as shown in Video S2. Figure 6c shows the experimental setup, the load hysteresis of the bearing pad and the voltage signals generated from the sensors. The developed AI model was used to predict the loads induced on the sensors. As shown, the proposed system array was successfully able to determine the loads induced on the bearing pad in real‐time.

FIGURE 6Potential applications of the proposed system a) Civil engineering applications such as bearing pads b) Mechanical engineering applications such as shock absorbers c) Demonstration of the proposed system as a self‐sensing bearing pad d) Demonstration of the proposed system as a self‐sensing shock absorber. Potential applications of the proposed system e) Biomedical applications such as neuromuscular rehabilitation equipment f) Demonstration of the proposed system as a patient‐specific smart hand gripper for neuromuscular rehabilitation with performance tracking features. g) Hierarchical arrangement for multi‐range force sensing h) Schematic diagram of a wireless signal transmission system for the proposed system.

**Figure:**
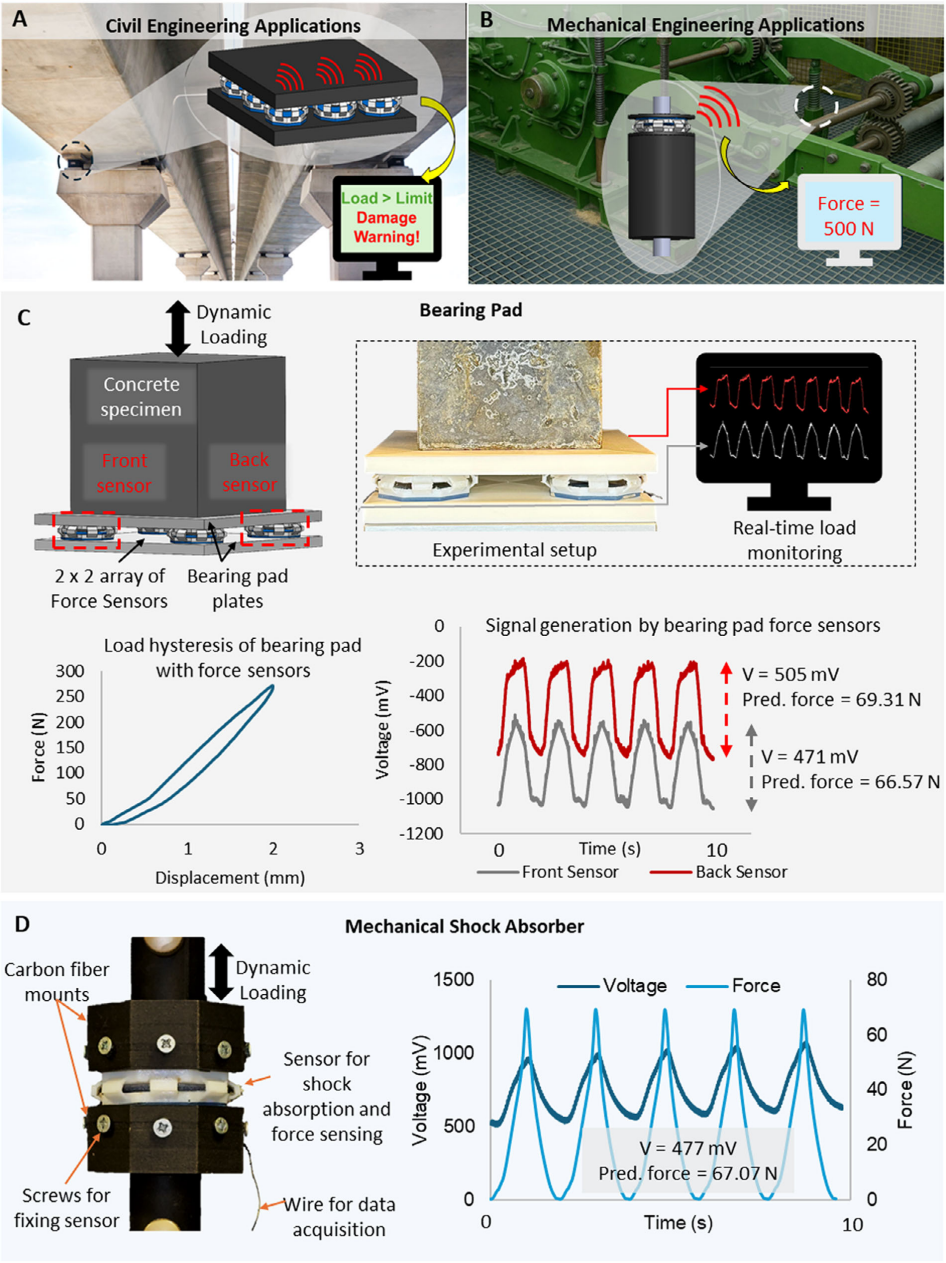

**Figure:**
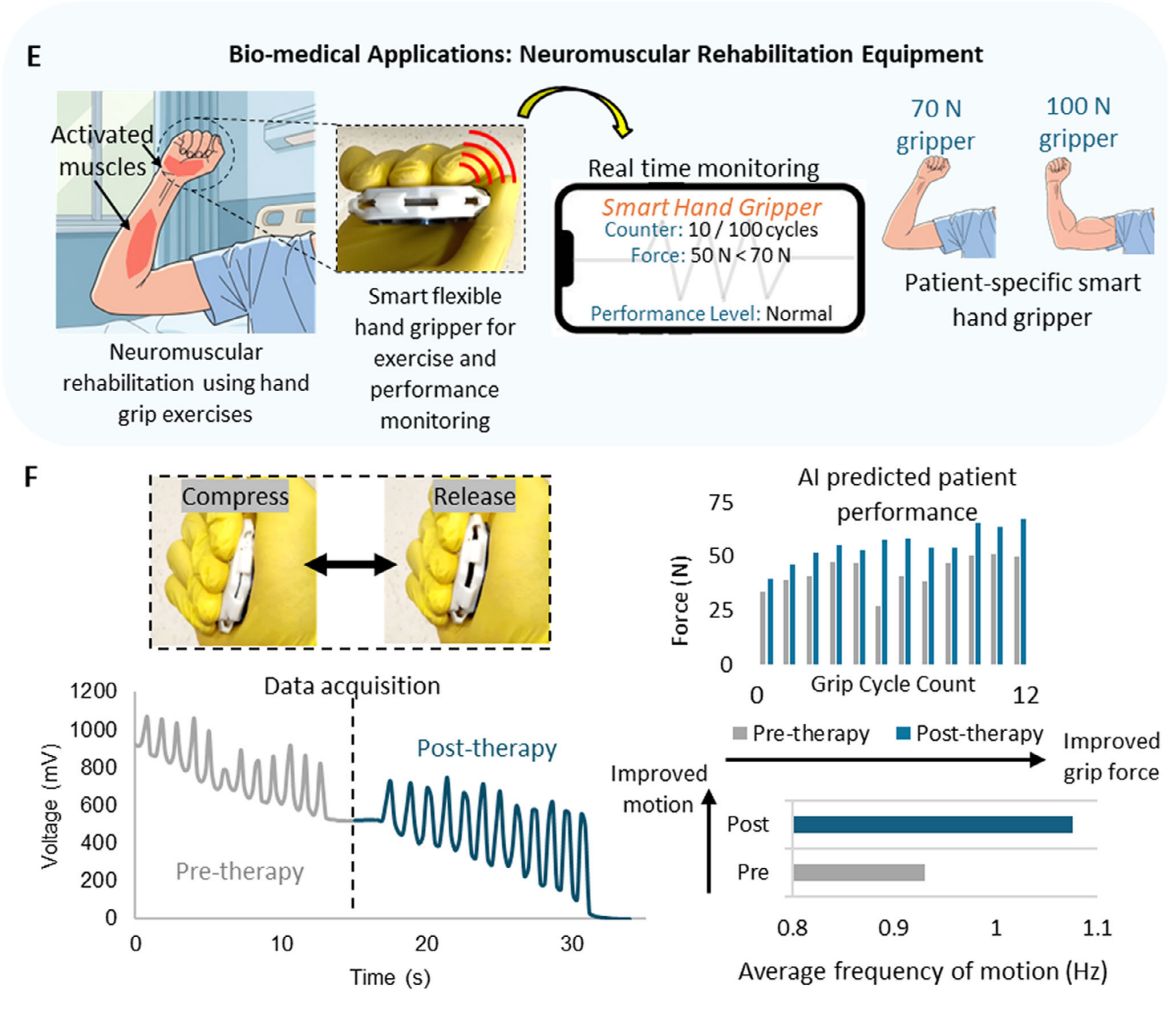

**Figure:**
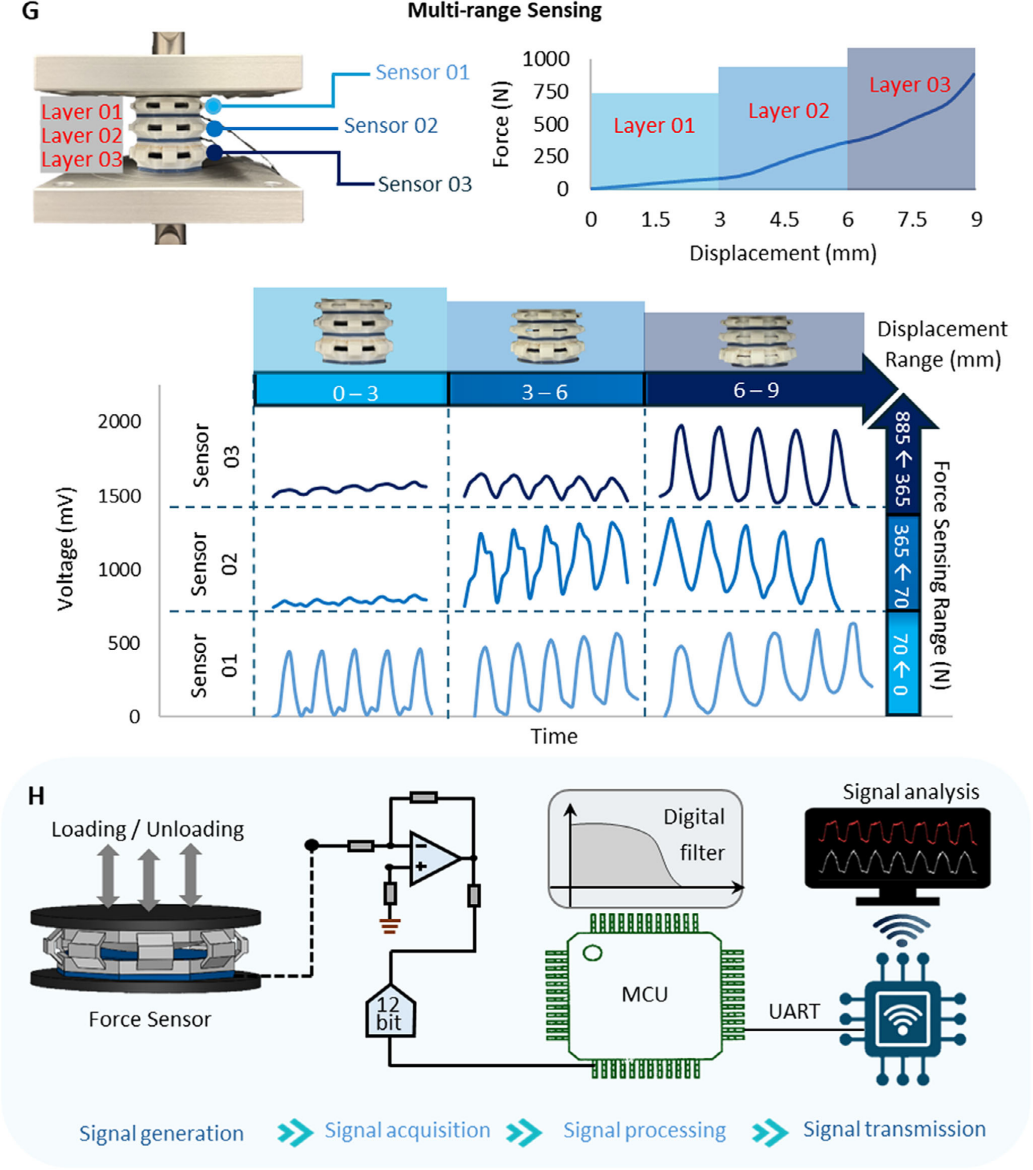

Similarly, it has the potential to be used in mechanical engineering applications such as mechanical shock absorbers in mechanical systems to simultaneously absorb shocks while monitoring the health of the system as shown in Figure 6b. Vehicular suspensions such as vibration isolation systems in vehicle seats for human comfort [85], precise equipment such as optical tables which require low‐frequency vibration isolation [86], ocean wave energy harvesters equipment [87] etc. are some potential applications in mechanical engineering. The applicability of the proposed system as a coil spring in shock absorbers is demonstrated in Figure 6d. The upper and lower mounts were fabricated using a carbon fiber reinforced polymer [CFRP] (Polyamide 12 – Carbon fiber). The fabricated system was mounted using screws and the component was dynamically loaded at a frequency of 0.5 Hz for a 3 mm displacement. As shown, the component was successfully able to under dynamic loading while monitoring the mechanical motion and quantifying the loads induced. A demonstration is shown in Video S3.

Beyond such sectors, the proposed system can be used in biomedical engineering applications as well as shown in Figure 6e. Many biomedical and human‐machine interfaces operate under the low‐frequency regime suggesting the strong potential of the proposed system for wearable devices, rehabilitation equipment etc. Specifically, we have demonstrated how this system can be used to develop patient‐specific neuromuscular rehabilitation equipment such as hand gripper to perform real‐time monitoring of the patient's activity such as the grip force, motion frequency and grip count while undergoing therapeutic exercises. The device can be tuned based on the patient's condition and muscular performance. Experimental tests were conducted using a (1,45) design as shown in Figure 6f. As shown, the device does real‐time monitoring throughout a patient's therapy period, and it displays results on the patient's performance by monitoring the frequency of motion and the grip force at a fixed number of grip cycles. The AI model effectively converts the electrical signals generated during the process to calculate these parameters.

Furthermore, a multi‐range force sensing strategy using different units is demonstrated as shown in Figure 6g. The strategy involves systematically arranging units hierarchically to enhance the force sensing range to monitor low and high forces without compromising the resolution and accuracy of the force measurements. This strategy implemented aims to address the limitations in current force sensing techniques to detect and quantify forces in a wide spectrum without the utilization of additional sensors. The implemented strategy demonstrates an enhanced force sensing system capable of monitoring low and high magnitude forces in a single‐integrated system. For example, such a system can monitor daily loads in an engineering system as well as unexpected high impact loads within a single‐integrated platform. As elaborated earlier, the force sensing range of system can be adjusted by systematically tuning key geometrical parameters such as t and Ө. By leveraging this tunability, distinct units can be developed, where each unit can be used to monitor forces in specific sub‐ranges in a wider spectrum. A hierarchical array of the units can be constructed to practically implement this. The performance of a 3‐layer array is shown in Figure 6g. The units are tuned to systematically measure 0 – 70 N, 70 – 365 N and 365 – 885 N using the first, second and third layers respectively. The utilized units are (1,45), (2,45) and (3,45) arranged from top to bottom in order.

In real world applications, the proposed system can be used as a multi‐functional force sensing system with wireless communication. A schematic diagram of a wireless signal transmission component for the proposed system is shown in Figure 6h. This system can comprise of the proposed system as the signal generation system, a TLC2201 amplifier to facilitate the signal acquisition, an Arduino MEGA 2560 unit to conduct signal processing and finally the signal transmission facilitated by an ESP32 unit. Owing to the closed‐form nature of the AI‐derived model, the force‐prediction equations can be readily embedded into microcontroller platforms such as the Arduino or ESP32. This enables on‐board, real‐time conversion of voltage signals into quantitative force outputs without requiring external computation hardware. As a result, the proposed system, together with the integrated AI model, can be seamlessly implemented in compact, low‐power, wireless sensing platforms suitable for field deployment.

## 3 Conclusions

In this study, we introduce a tunable meta‐nanogenerator‐based sensory system in a plug‐and‐play manner for multi‐range force measurement. The proposed force sensing system is a tunable meta‐tribo system designed to accurately sense and quantify forces induced on the material system. The geometrical parameters of the MM design can be effectively tuned to vary the force sensing range of the sensor based on the requirements and application. The proposed system can be tuned to even surpass the force sensing ranges of existing triboelectric sensors. We carried out theoretical, numerical and experimental studies to validate the performance of the proposed system for load bearing and sensing. Since the loading criteria such as the deformation and operating frequencies can vary based on different applications, we have also demonstrated the capability of the sensory system to generate unique electrical signals for different scenarios. Advance AI techniques were employed to couple the generated electrical signals and the forces with high accuracies. The relationships are demonstrated in functional forms to facilitate the use of the proposed system in practical applications. The versatility of the proposed system is demonstrated through civil, mechanical and biomedical engineering applications, displaying its reliability for simultaneous load bearing and real time self‐sensing applications. Furthermore, force range enhancement and multi‐range force sensing in a single integrated system are demonstrated by designing hierarchical arrays of the proposed system. The proposed method displays promising results for future smart and self‐sensing engineering systems. Future directions would be performing more experiments to create a larger dataset to further enhance the reliability and robustness of the system. Furthermore, the proposed system shall be further evaluated under higher‐frequency loading conditions, long‐term fatigue and environmental variables such as humidity to fully assess its performance in broader real‐world scenarios. In addition to that, with the development of advanced manufacturing techniques, the design shall be further tested with other materials to enhance the TENG system to generate high resolution signals and also to suit a wider range of engineering applications.

## 4 Materials and Methods

### 4.1 Numerical modeling

Numerical modelling to analyze the mechanical behavior of the proposed system was done by Finite Element Analysis (FEA) using the COMSOL Multiphysics software. A 3D solid mechanics physics interface was used for all cases. The material non‐linearity of TPU and TPE was captured in the simulations by considering the materials as hyperelastic using a two‐parameter Mooney‐Rivlin model. Numerical modelling of the TENG was also done using COMSOL. Specifically, the “electrostatics” and “electrical circuit” interfaces in the AC/DC module were used. A 2D interface was used to reduce the computational time and complexity of the simulation. Refer Section S4 for more information on numerical modelling of the mechanical behavior and TENG.

### 4.2 Fabrication of Elements and Experimental Methods

All fabrications were carried out using additive manufacturing technologies. The TPU, TPE and CFRP parts were fabricated using dual extruder 3D printers. Specifically, the Raise3D Pro3 and Raise3D E2CF printers were used for this purpose. All the 3D CAD models were created using the Dassault Systèmes SolidWorks software. The loading experiments were conducted using the ADMET 8610 testing machine equipped with a 5 kN load cell. The voltage signals generated from the proposed system were measured using a National Instrument 9220 DAQ module. LabView programs were developed to control the entire electrical signal data acquisition system. Temperature controlled tests were conducted with an environmental chamber equipped to the ADMET 8610 testing machine. Refer Section S6 for more information on the material properties and experimental methods.

## Author Contributions

Q.Z. conceived the concept. R.P. and Q.Z. designed and performed the experiments. R.P. and S.G. carried out the fabrications. R.P., P.A., and Q.Z. contributed to the design of the relevant device and experiments, and interpretation of the data. All authors analyzed and interpreted the data. P.J supervised the research. R.P. and Q.Z. prepared the manuscript draft. All authors contributed to editing the manuscript.

### Conflicts of Interest

The authors declare no conflicts of interest.

## Supporting information




**Supporting File 1**: advs73870‐sup‐0001‐SuppMat.docx.

**Supporting File 2**: advs73870‐sup‐0002‐Video S1_Revision_1.mp4.

**Supporting File 3**: advs73870‐sup‐0003‐Video S2_Revision_1.mp4.

**Supporting File 4**: advs73870‐sup‐0004‐Video S3_Revision_1.mp4.

## Data Availability

The data that support the findings of this study are available from the corresponding author upon reasonable request.
