# Supplementary material for: Tunable Plug‐and‐Play Meta‐Nanogenerator Materials for Multi‐Range Force Measurements
Source: Adv Sci (Weinh). 2026 Jan 20;13(17):e00009. doi: 10.1002/advs.202600009 (PMC13042629; doi:10.1002/advs.202600009)
Supplement: Supplementary file 1 — Supporting File 1: advs73870‐sup‐0001‐SuppMat.docx. [file ADVS-13-e00009-s001.docx]

**Supporting Information**

**Tunable Plug-and-Play Meta-Nanogenerator Materials for Multi-range Force Measurements**

**Roshira Premadasa^1^, Pouya Almasi^1^, Samriddhi Ghimire^1^, Wenkui Dong^2^, Chenjie Zhang^3^, Pengcheng Jiao^3*^, Qianyun Zhang^1*^**

*^1^Department of Civil Engineering, New Mexico State University, Las Cruces, NM, USA*

*^2^Centre for Infrastructure Engineering and Safety, School of Civil and Environmental Engineering, The University of New South Wales, NSW, Australia*

*^3^Ocean College, Zhejiang University, Zhoushan, Zhejiang, China*

# S1 Literature Review

***Table S1:*** *Evaluation metrics used for semi-quantitative scoring of previous meta-tribo work*

| **Score** | **Quantitative Force Sensing** | **Mechanical Tunability** | **Multi-range Measurement** | **Modularity** | **Fatigue Resistance** | **Multifunctionality and Versatility** |
| --- | --- | --- | --- | --- | --- | --- |
| **0** | Only qualitative trends (voltage increases with force), no calibration curve. | No tunability; single fixed geometry. | Single, narrow operating range. | Fully monolithic; sensing and structure cannot be separated. | No cycling or stability data. | Self-powered sensing capability in one domain |
| **1** | Single calibration curve, but low R² or no error reporting. | Only minor geometric variation explored; no systematic analysis. | Slightly extended range, but still essentially one regime. | Some replaceable parts but sensing layer strongly integrated. | Poor short-term mechanical fatigue performance demonstrated (<10^2^ cycles) | Self-powered sensing + 1 functionality, but not demonstrated across domains |
| **2** | Basic calibration with noticeable error; shows quantitative intent but not reliable or repeatable. | Minor tunability with unclear effect; geometry changed but no quantified or modeled relationship | Two ranges appear, but not intentionally engineered | Some replaceable parts, but removal changes functionality or integration is weak | Short-term fatigue (~10²–10³ cycles) with noticeable degradation. | Self-powered sensing + min 2 functionalities, in one domain |
| **3** | Reasonable calibration (e.g., R² > 0.95) in a single range, no model beyond simple fitting. | One tunable geometric parameter (e.g., thickness) with clear trend. | Two distinguishable ranges (e.g., low vs high force) but not explicitly engineered. | Partially modular (e.g., replaceable electrodes or layers). | Good short-term mechanical or electrical fatigue performance demonstrated (<10^3^ cycles) | Self-powered sensing + min 2 functionalities in two domains |
| **4** | Good calibration over a wide range, with error metrics (R², MAE) and independent validation. | Two or more parameters tuned with theory/FEA support. | Clearly engineered multiple ranges with different modules | Clear modular blocks with some independent replacement. | Good short-term mechanical and electrical fatigue performance demonstrated (<10^3^ cycles) | Self-powered sensing + 2 functionalities in two domains |
| **5** | Full quantitative framework (analytical / AI model) with low error, validation, and uncertainty analysis. | Systematic multi-parameter design space (theory + FEA + experiments) showing large, controllable stiffness/range changes. | Hierarchical / modular architecture with seamless low–mid–high range measurement in one platform. | True plug-and-play: mechanical and electrical components are independently fabricated, replaced, and reconfigured. | >10⁵ cycles with minimal degradation for mechanical/electrical fatigue | Exhibits multiple inherent functions (e.g., load-bearing, energy absorption, quantitative self-powered sensing), demonstrated across at least three distinct domains. |

# *Table S2: Comparison of reported triboelectric and meta-triboelectric force sensing systems relative to this work.*

| **Study** | **Concept** | **TENG mode / Materials** | **Quantitative Force Sensing** | **Tunable** | **Multi-range Sensing** | **Modularity** | **Multifunctional** | **Mechano-Electrical Coupling** | **Maximum Force Sensing Range (kPa)** | **Sensing Accuracy (%)** | **Sensitivity** |
| --- | --- | --- | --- | --- | --- | --- | --- | --- | --- | --- | --- |
| Rasel et al., 2018 | Triboelectric | *CS /PDMS - PDMS CNT* | **✅** | ❌ | ❌ | ❌ | ❌ | N/A | 200-450 | N/A | 0.04 V/kPa |
| Qin et al., 2024 | Triboelectric | SE /  GBM IR PDMS - FEP | **✅** | ❌ | ❌ | ❌ | ❌ | Demonstrated (Basic linear regression) | 5-1240 | 99.8% | 0.023 V/kPa |
| Tao et al., 2020 | Meta-Triboelectric | CS /  Kapton-PLA | ❌ | ❌ | ❌ | ❌ | **✅** | N/A | - | N/A | N/A |
| Barri et al., 2021 | Meta-Triboelectric | CS /  TPU-PLA | ❌ | ❌ | ❌ | ❌ | **✅** | N/A | N/A | N/A | N/A |
| Xu et al., 2021 | Meta-Triboelectric | CS /  PTFE-Al | ❌ | ❌ | ❌ | ❌ | **✅** | Demonstrated  (Basic linear regression) | - | N/A | N/A |
| Barri et al., 2022 | Meta-Triboelectric | CS /  TPU-PLA | ❌ | ❌ | ❌ | ❌ | **✅** | N/A | N/A | N/A | N/A |
| Chen et al., 2025 | Meta-Triboelectric | S-CS /  PTFE-Cu | ❌ | ❌ | ❌ | ❌ | **✅** | N/A | - | N/A | N/A |
| **This Work** | **Meta-Triboelectric** | **SE /**  **TPU-Ag** | **✅** | **✅** | **✅** | **✅** | **✅** | **✅ (AI)** | **15-1600 and beyond** | **99.8%** | **0.29 mV/kPa** |

#

# S2 Mechanical Performance of the Proposed System

***S2.1 Theoretical Force-Displacement Relationship of the Proposed System***

For a single arm in the unit cell (Figure S1), the moment (M) at the fixed end of the arm can be defined as;

***
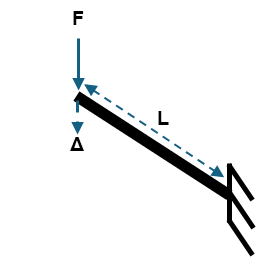
Figure S1:*** *Free body diagram of a single arm of the stiffness element*

$M=\left( \frac{3EI}{L^{2}} \right)x \Delta$ (S1)

here;

E = Modulus of Elasticity of the material,

b = width,

t = thickness of the arm

Ө – angle of the arm

L = length of the arm; $L=\frac{2.3}{sin(Ө)}+t$

Δ = vertical deformation at the free end

$I= \frac{1}{12}bt^{3}$ ; I = 0.0833bt^3^

But,

M = F x L cos(Ө) (S2)

where, F = force exerted on the arm during deformation Δ

By Equations S1 and S2,

$F=\left( \frac{3EI}{L^{3}cos(Ө)} \right)x \Delta$

Since there are 8-sides in the mechanical metamaterial (MM);

$F=\left( \frac{3EI}{L^{3}cos(Ө)} \right)x 8\Delta$ (S3)

By substituting E, I and L;

$F=\left( \frac{2bEt^{3}}{{(\frac{2.3}{Sin\left( Ө \right)}+t)}^{3}cos(Ө)} \right)x \Delta$ N (S4)

In this study, the maximum deformation of the mechanical component (MC) is limited to 3 mm.

Therefore, the maximum force (F_max_) on the MC is;

$F_{max}=\left( \frac{6bEt^{3}}{{(\frac{2.3}{Sin\left( Ө \right)}+t)}^{3}cos(Ө)} \right)$ N (S5)

and the compliance / sensitivity (S) of the MC is;

$S=1/\left( \frac{2bEt^{3}}{{(\frac{2.3}{Sin\left( Ө \right)}+t)}^{3}cos(Ө)} \right)$ mmN^-1^ (S6)

Where the sensitivity-S here is the deformation per unit force.

***S2.2 Mechanical Behavior and Energy Absorption of the Proposed System***

As discussed in the main paper, the mechanical behavior can be tuned. Figure S2a demonstrates the force-displacement behavior of the (5,55) design for b = 20 mm obtained using numerical simulations and experiments. The design achieves a maximum force of 3297 N (1707 kPa) which is further tunable. As discussed in the main paper, the MC of the proposed system is fabricated using Thermoplastic Polyurethane (TPU-95A). While not limited to this material, the proposed system can also be fabricated using other materials as well, to cater to different applications and the required mechanical performances. To demonstrate its versatility, the MC was fabricated using different materials that possess different stiffnesses. As shown in Figure S2b, the force-displacement relationships were experimentally obtained for units fabricated using TPU 95A, PA12-CF, PLA and metal (AlSi10Mg). More details on the materials and fabrication methods are given in Section S6. The blue markers show the maximum operating range of the sensor which signifies the elastic range, or in the case of the TPU sample, the design’s deformation limit.

The system was also experimentally tested to verify the negligibility of the electrical component (EC) on the performance of the MC. An experimental test was conducted on the MC with and without the EC attached. The experiment was conducted on a (1,45) TPU sample. As shown in Figure S2c, although the test with EC showed higher force results, the maximum difference between the readings was approximately 2% indicating the EC does not have a significant effect on the performance of the MC.

The design also demonstrates excellent energy absorption capabilities, indicating its suitability for a wide range of engineering applications including safety barriers, impact mitigation systems and other smart load bearing applications. As shown in Figure S2d, the energy absorption density (W) for different designs is demonstrated. The W was calculated by the area under the respective experimental force-displacement curves and divided by the bulk volume. The energy absorption capability is completely tunable as well. The (3,45) unit alone has a high W of 454 kJ/m^3^. The plots for the maximum forces along with error bars are given in Figure S2e-f to demonstrate the repeatability of the measurements.


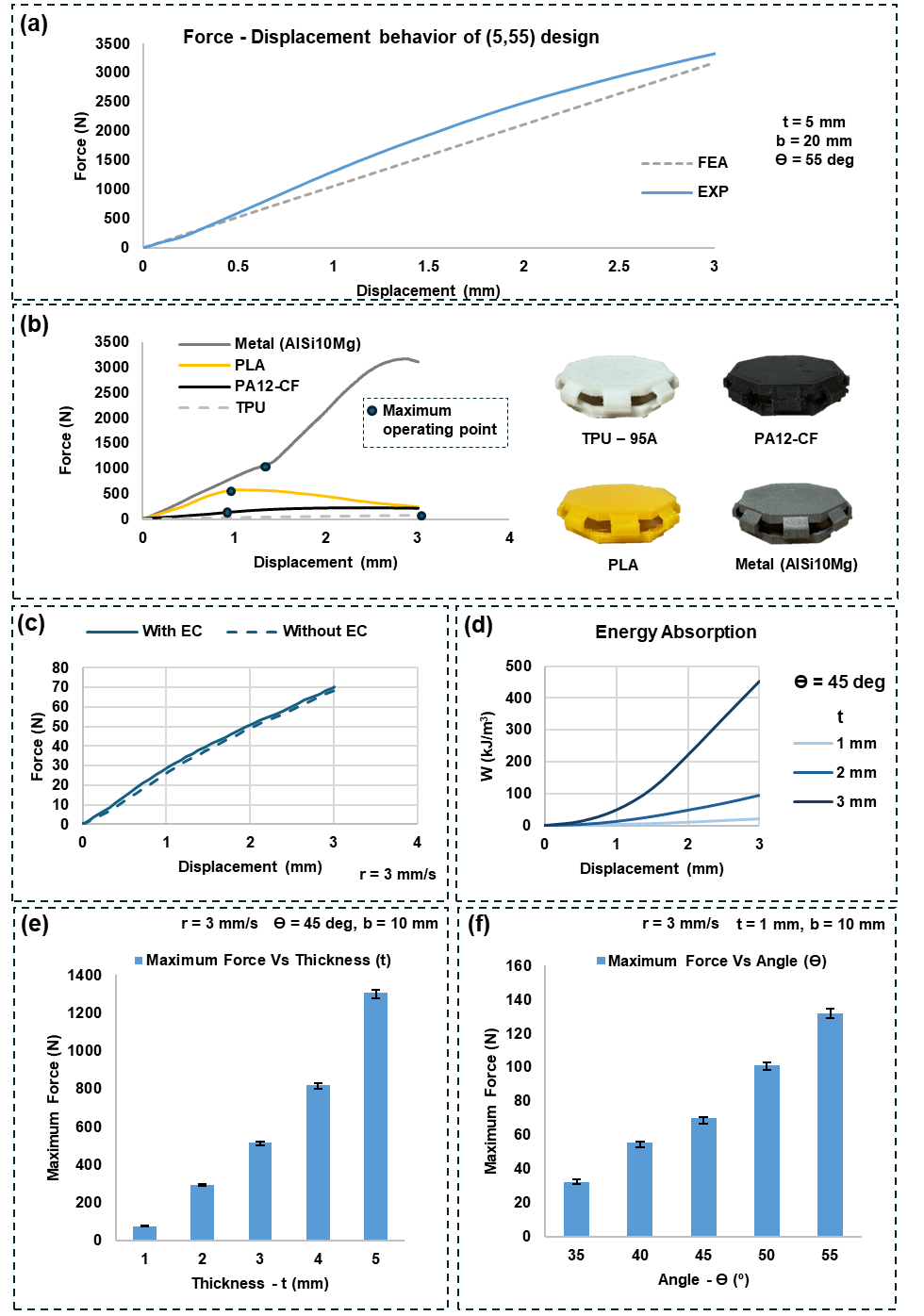


***Figure S2:*** ***Mechanical behavior of the proposed system*** *a) Force-displacement behavior of (5,55) design* *b) Force-displacement curves with different materials c) Influence of the electrical component for mechanical behavior d) Energy absorption performance based on experimental data. Bar charts including error bars demonstrating the repeatability of the measurements for e) Maximum force vs thickness (t) f) Maximum force vs angle (*$Ө$*).*

# S3 Electrical Performance of the Proposed System

***S3.1 Contact Area Evolution of TENG***

The varying contact area can be described in relation to the change in the surface area of the dome when it penetrates by a distance x into the electrical component. The relationship between the contact surface area (A_x_) and the penetration depth x can be derived as follows.

For the dome; radius (r) = 21 mm and height (h) = 4.5 mm

Radius of curvature (R_C_) = $\frac{r^{2}+h^{2}}{2h}$ = 51.25 mm

The radius of the dome at a depth x from the top (r_x_) = $\sqrt{{R_{C}}^{2}-\left( R_{C}-x \right)^{2}}$ = $\sqrt{{51.25}^{2}-\left( 51.25-x \right)^{2}}$ mm

Hence, surface area of the dome at depth x from the top (A_x_) = $\pi(x^{2}+{r_{x}}^{2})$

A_x_ = $102.5\pi x$ mm^2^ (S7)

***S3.2 Experimental Behavior of Single-Electrode Mode TENG***

The open circuit voltage (V) and short circuit current (I) generated by the proposed system at a displacement of 3 mm is shown in Figure S3a. In these testing conditions, the sensor generates a peak V and I of 475 mV and 0.29 nA respectively. The voltage output signals with error bars demonstrating repeatability across multiple signals is shown in Figure S3b.

The variation in the voltage signals with different frequencies ranging from 0.5 Hz to 2.5 Hz was also explored as shown in Figure S3c. As it can be seen, although incomplete charge transfer occurs at higher frequencies, the sensor is capable of generating unique signals for force predictions at even higher frequencies.

To verify whether the reduction in electrical signals occurs due to incomplete contact area, an experimental contact-area imprint test was conducted utilizing a silver-ink transfer method as shown in Figure S3d. Silver ink was applied to the dome of the MC and was imprinted on blue tape attached on the EC. The test was conducted at a low (0.5 Hz) and high (1.5 Hz) frequency. The imprints were overlaid with contact-area contour plots generated using FEA. The real contact-area remained approximately unchanged at both low and high frequencies indicating that the high frequencies don’t hinder surface conformity at deformation. However, as shown in Figure S3d, the reduction in voltage signals at high frequency closely aligns with the reduction in contact time (time taken to reach maximum deformation and to return to original position ≈1/f) which shortens the time for complete charge transfer. The analysis confirms that the voltage decay arises from insufficient contact time, rather than loss of contact area.


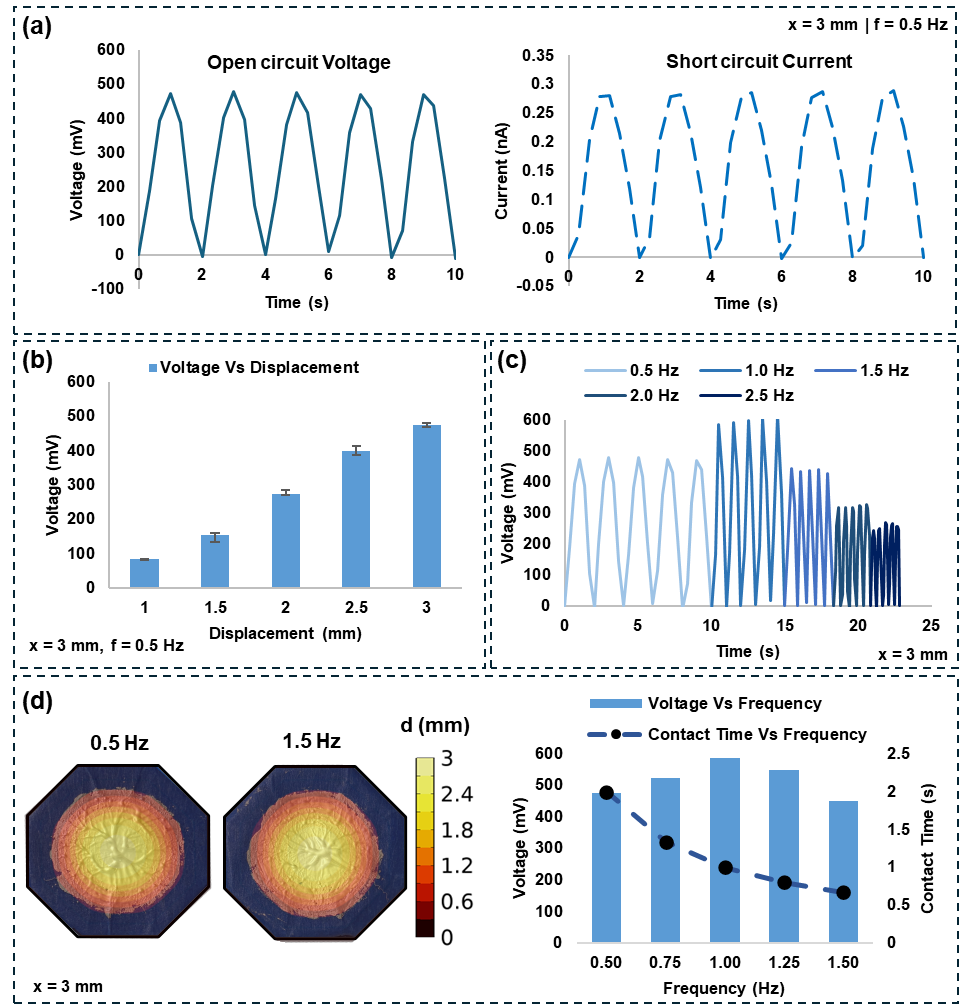


**Figure S3:** **Electrical performance of the proposed system** a) Open circuit voltage and short circuit current generation in experiments b) Voltage output signals with error bars demonstrating repeatability across multiple signals c) Voltage signal behavior at 0.5 Hz to 2.5 Hz operational frequencies d) Contact area and time analysis of proposed system at low and high frequencies.

**S4 Numerical Modelling**

***S4.1 Numerical Modelling of Mechanical Behavior***

Numerical modelling to analyze the mechanical behavior of the proposed system was done by Finite Element Analysis (FEA) using the COMSOL Multiphysics software [1]. Specifically, the “non-linear structural materials” module with the “solid mechanics” module was used. A 3D solid mechanics physics interface was used for all cases.

The material non-linearity of TPU was captured in the simulations by considering the material as hyperelastic using a two-parameter Mooney-Rivlin model, which particularly is used for such soft polymers. The Mooney-Rivlin parameters were obtained by fitting the model to the experimental stress-strain data obtained by performing uniaxial tests for the materials. Material properties are given under Section S6.

The CAD model was imported to COMSOL in the form of a .step file and discretized using a free tetrahedral mesh. Localized mesh refinements were enabled to accurately capture high strain gradients. The assigned boundary conditions were a fixed boundary condition at the base and a prescribed displacement at the top surface to exactly mimic the experimental setup. A parametric sweep for the prescribed displacement parameter was done to capture the entire displacement-controlled force-displacement behavior. A mesh convergence study was conducted from a course to finer mesh until the response force variation was less than 5%. The Von-Mises stresses generated in the numerical simulation for the (1,45) sample are shown in Figure S4.

***
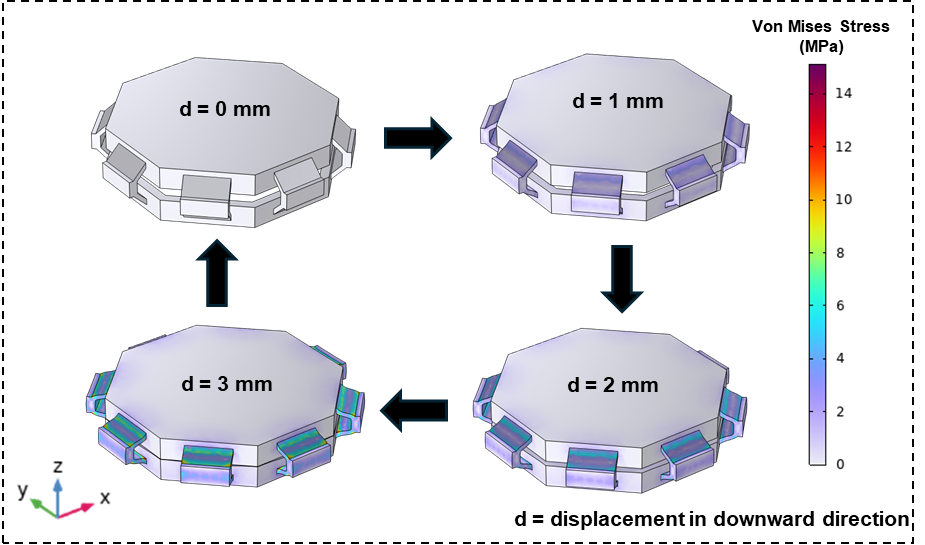
***

***Figure S4: Numerical modelling of mechanical behavior of the proposed system*** *– Von Mises stresses in numerical simulations*

***S4.2 Numerical Modelling of TENG***

The COMSOL Multiphysics software was used to perform the numerical modelling of the TENG. Specifically, the “electrostatics” and “electrical circuit” interfaces in the AC/DC module were used. A 2D interface was used to reduce the computational time and complexity of the simulation.

Initially a ground condition was assigned to the boundaries of a bounding box created around the modelling space to simulate an air box condition with approximately zero electric potential at the boundaries. This leads to effective convergence of the simulations. Since the key factor in this simulation is the contact surface area, the 3D behavior was effectively captured in the 2D interface by changing the in-plane depth parameter of the geometry. This technique also reduced the level of complexity in the simulation.

In order to perform multi-physics coupling between the electrostatics and electrical circuit interfaces, external I vs U coupling was done by assigning the electrodes as terminals. The open circuit voltage was measured using the difference between the electrical potentials of the electrode and the reference electrode. Finally, a time dependent study was conducted using the displacement rate. All parameters that were used for the simulation are given in Table S3. The V-Q-x behavior of the proposed system obtained using FEA is given in Figure S5.

***Table S3:*** *Parameters used for numerical modelling of TENG*

| **Geometrical Properties** | Length | 42 mm |
| --- | --- | --- |
|  | In-plane depth (3 mm displacement) | 6.14 mm |
|  | Thickness of dielectric 1 | 0.4 mm |
|  | Thickness of dielectric 2 | 0.1 mm |
|  | Maximum gap | 3 mm |
| **Material Properties** | Relative permittivity of the  dielectric layer (TPU) | ε_r1_ = 7.2 |
|  | Permittivity of vacuum | ε_0 =_ 8.854 x 10^-12^ |
|  | Surface charge density | σ = 75 x 10^-6^ C/m^2^ |
| **Other Properties** | Rate of displacement | 3 mm/s |

The V-Q-x relationship for the proposed system is as shown in the figure below.


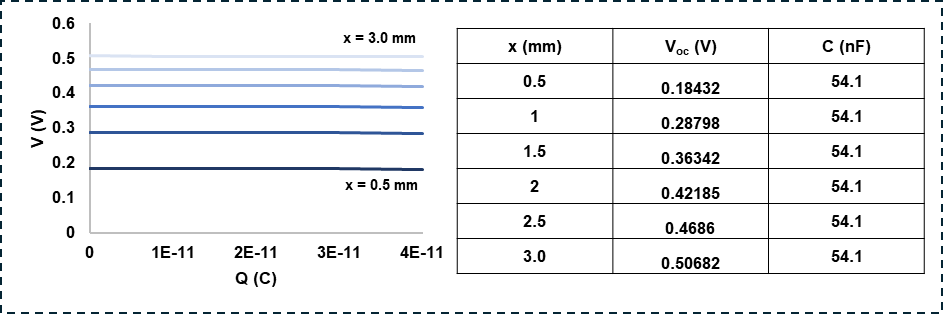


**Figure S5:** V-Q-x behavior of the proposed system

**S5 System Integration**

***S5.1 AI-Driven Mechano-Electrical Coupling***

To perform the system integration by mechano-electrical coupling, an artificial intelligence (AI) driven approach is implemented in this study. Rather than utilizing a traditional physics-based model which usually struggles to capture highly non-linear and unpredictable behaviors in the relationship between a sensor’s inputs and outputs using analytical and empirical formulas, a data driven-AI approach is utilized in this study which effectively captures the hidden relationships in either operational or experimental data. Such an approach is essential, especially in sensor applications where the effect of practical aspects need to be integrated. Specifically, a symbolic data mining approach using evolutionary AI is utilized, which unlike other data mining approaches/or black box machine learning models, is capable of unveiling the hidden relationships between the input and output parameters through a symbolic equation, which enables easy system integration and optimization for real-world applications. Rather than utilizing a traditional single-gene genetic programming approach which often struggles with overfitting with highly nonlinear data due to the reliance on a single tree structure to capture the nonlinearity, a multi-gene variant of genetic programming (MGGP) is used which evolves multiple genes that each individually captures distinct non-linearities in the sensor and is later combined based on their weights after the evolutionary process. The MGGP initiates with a set of candidate models in a population, each consisting of multiple genes as trees. The maximum number of genes and tree depth parameters in the program controls complexity and computation power. These models compete in tournaments over generations, and the best performing models are chosen for reproduction, where mutations and crossovers occur. The process continues over generations and reveals the best performing model.

In this study, the MGGP is implemented using the GPTIPS2.0 program [2]. Two models were trained using the program, which were later fused together. The first model (Model 1) was used to capture the relationship between the electrical signals and the deformations. The voltage (V) and frequency (f) of the electrical signals were used as input parameters to predict the displacement (d) undergone by the proposed system. A graphical representation of the experimental data used is shown in Figure S6. The best performing model is shown in Equation S8. This is a global model that can be implemented to any design.

$d=0.00438V -f(0.139+1.01cos\left( f \right))$ + 1.26 (S8)

d = displacement (mm), V = voltage (mV), f = frequency (Hz)

A second model (Model 2) was trained to predict the force (F) values using the input parameters, displacement (d) and the rate of displacement (r). For demonstration, experimental data for the (1,45) design was used. Force-displacement data for different displacement rates from experiments were used to train the model. The best performing model is shown in Equation S9.

$F_{(1,45)}=\frac{d}{r}\left( 0.315r^{2}+30.9r-2.48dr - 2.79 \right)-0.364$ (S9)

F = force (N), d = displacement (mm), r = 2df [displacement rate (mm/s)]

Now by fusing Model 1 and Model 2, a global model is created for the (1,45) design where the force values are predicted effectively by using the data from electrical signals.

The input parameters used and the generated gene tree structures for the models are shown in Table S4 and Figure S7 respectively.

**
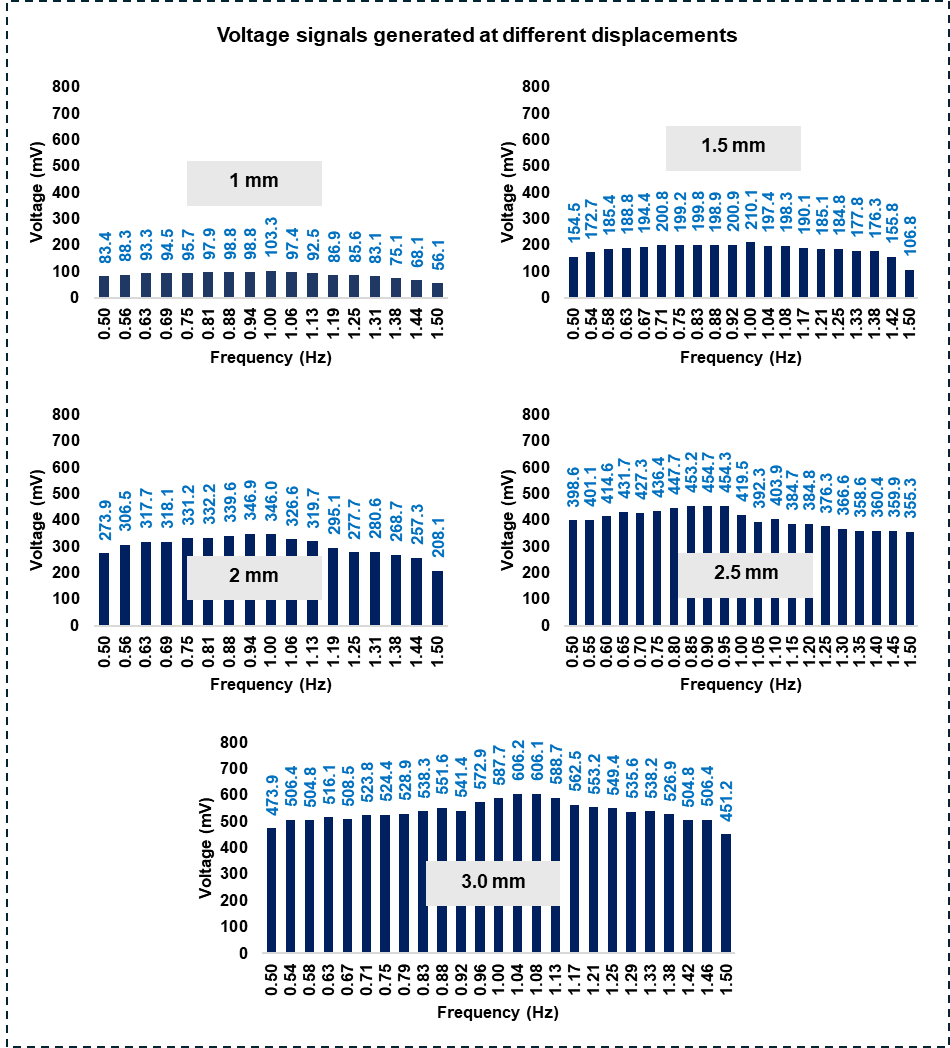
**

**Figure S6:** Voltage signals generated by the proposed system at various frequencies and displacements

***Table S4:*** *Input parameters of AI algorithm for Model 1 and Model 2*

| **Input Parameter** | **Model 1** | **Model 2** |
| --- | --- | --- |
| ***Population Size*** | 25 | 25 |
| ***Maximum Generations*** | 1500 | 1500 |
| ***Tournament Size*** | 25 | 25 |
| ***Maximum genes*** | 2 | 3 |
| ***Maximum Tree Depth*** | 3 | 3 |
| ***Function Set*** | Times, Minus, Plus, Divide, Square, Cube, Cosine | Times, Minus, Plus, Divide, Square, Cube, Cosine, Sine |

**
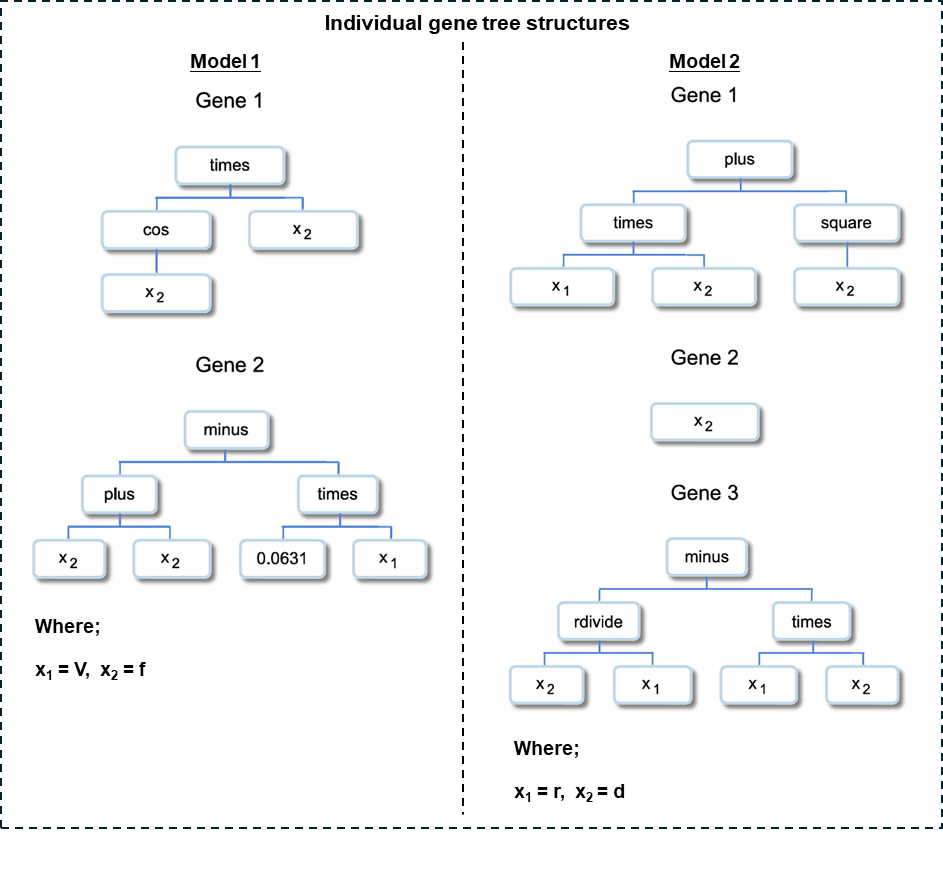
**

**Figure S7:** AI algorithm generated gene trees of models 1 and 2

Similarly, models for other designs can be trained seamlessly. For demonstration, the generated models for the (2,45) and (3,45) designs are shown in Equation S10 and Equation S11 respectively. Both models displayed high accuracy with R^2^ = 0.99 for all training, validating, and testing data.

$F_{(2,45)}=d\left( 3.72d^{2}-51.5d+247 \right)-\frac{30.8d^{2}}{r+d}-5.3$ (S10)

$F_{(3,45)}=d\left( 2.8d^{2}-51.1d+1.6r-\frac{20.4}{r}+311 \right)-0.995$ (S11)

Where d = Equation S8 and r = (Equation S8) x f

***S5.2 Analysis of Model Uncertainty***

**
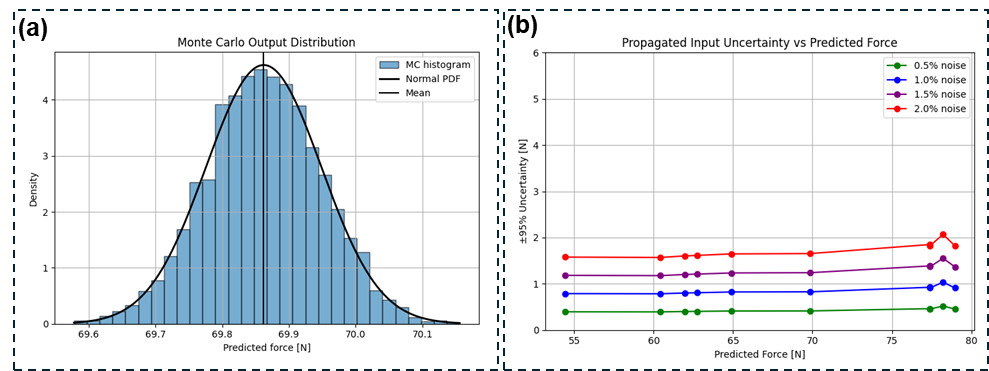
**

**Figure S8:** (a) Monte Carlo simulation of the closed-form model showing the distribution of predicted forces under input noise perturbations, with the output following a normal distribution. (b) Propagated input uncertainty for different noise levels (0.5–2.0%), indicating that the model remains stable with low sensitivity to input fluctuations across the predicted force range.

To assess the robustness and reliability of the closed-form predictive model, an uncertainty analysis was performed by introducing controlled perturbations to the model inputs and evaluating how these variations propagate to the predicted force outputs. Specifically, the model was initially assessed using the Monte-Carlo method [3], a method which evaluates model robustness by repeatedly sampling inputs with randomized perturbations, was used to quantify how measurement noise propagates to the predicted force outputs. The baseline voltage noise of the TENG output was measured as ≈ 0.1 mV under steady-state conditions. To evaluate robustness, Monte-Carlo simulations were repeated using a conservative noise level of 1 mV (10× the measured noise). For the representative test point analyzed, the Monte-Carlo simulation produced the distribution as shown in Figure S8a. The Gaussian fit closely matches the simulated distribution, indicating that within this region of the input space, the model responds in a smooth manner to input perturbations. The width of the distribution, on the order of only a few tenths of a newton, confirms that the voltage and frequency noise used in the experiment have a limited ability to distort the predicted force.

While the single-point Monte-Carlo analysis describes how measurement noise affects the predicted force at one operating condition, it does not reveal how this sensitivity varies across the full range of voltages, frequencies, and force levels present in the dataset. To address this, a point-wise uncertainty propagation was performed for every sample in the test set. This produces an uncertainty curve that shows how the input-induced uncertainty changes as a function of the predicted force, providing a global view of the model’s sensitivity to sensor noise. For each data point i, the measured voltage and frequency were perturbed many times using the same Gaussian noise model described earlier with noises ranging from 0.5%-2.0%. Plotting ${Confidence Interval}_{95\%, i}$ against the predicted force $F_{predicted}, i$ produces an uncertainty curve representing the noise sensitivity across the operating domain. As shown in Figure S8b, the resulting curves follow the expected behavior for a nonlinear model with mild local variations in sensitivity. Across the entire dataset, the input-induced uncertainty remains extremely small, with typical 95% intervals typically ranging from ±0.4 N to about ±2 N, depending on the operating point and the applied percentage noise level. These values are consistent with the single-point Monte-Carlo results and reflect the weak influence of voltage and frequency noise on the predicted force.

***S5.3 Parametric Analysis of Closed-Form Model Predictions***

Figure S9 presents a parametric analysis to validate that the closed-form model accurately reflects the underlying mechano-electrical coupling observed experimentally.

**
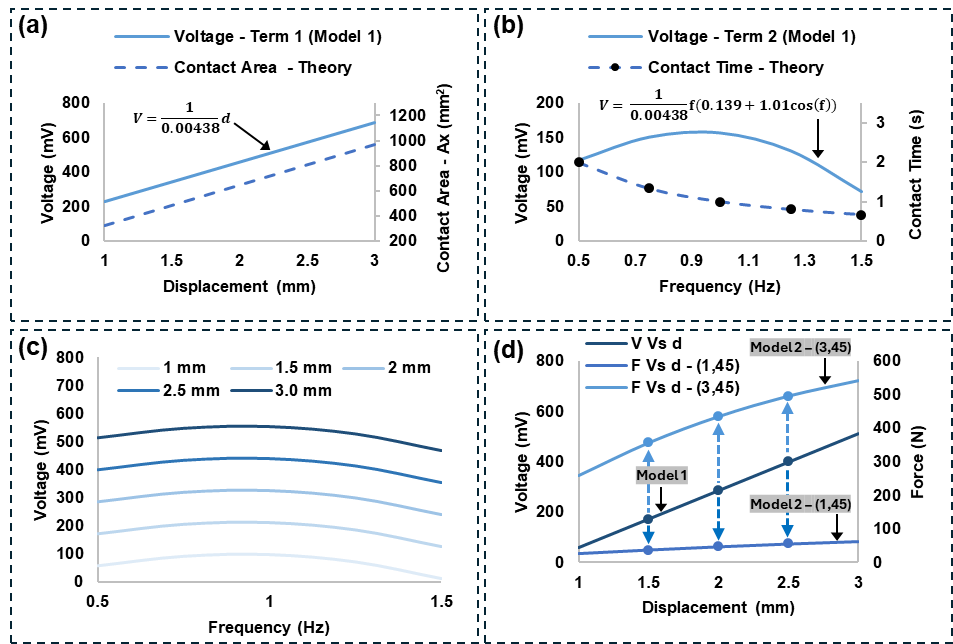
**

**Figure S9:** **Parametric Analysis of Closed-Form Model Predictions** a) Voltage-displacement behavior captured by term 1 in Model 1 (Behavior is proportional to the contact area evolution) b) Voltage-frequency behavior captured by term 2 in Model 1 (Incomplete charge transfer displayed at higher frequencies due to lesser contact time) c) Voltage-frequency behavior at different displacements in Model 1 (Model captures unique signals at different displacements regardless of operating frequency) d) Mechano-electrical coupling performed by fused model to predict forces at particular displacements (Model 2 developed for two different designs with t = 1 and t = 3 plotted against Model 1)

The first term of Model 1 shows that voltage increases with displacement in a manner consistent with deformation-driven contact area growth as shown in Figure S9a. The modeled voltage trend follows the same trajectory as the theoretical prediction of contact area evolution, confirming that the closed-form expression captures the deformation-dependent triboelectric response. The second term of Model 1 predicts the non-monotonic voltage variation with frequency as shown in Figure S9b. The model reproduces the reduction in voltage at higher frequencies, matching the theoretical decrease in contact time. This agreement confirms that the model correctly represents the charge transfer limitation caused by shorter contact duration at higher frequencies.

The model outputs distinct voltage–frequency signatures for multiple displacement levels (1–3 mm) as shown in Figure S9c. Despite having different deformation amplitudes, the curves exhibit a similar frequency dependence, demonstrating that the model consistently captures how frequency affects charge transfer across different operating conditions.

Model 2 for the (1,45) and (3,45) designs is evaluated against Model 1 here to show how the electrical output can be translated into force at different deformations and frequencies as shown in Figure S9d. The predicted force–voltage curves shift with deformation, confirming that the model identifies unique mechano-electrical mappings at each displacement condition. This demonstrates that the fused model is capable of predicting force from voltage signals under varying deformation inputs.

**S6 Material and Experimental Methods**

***S6.1 Materials***

The base material of the MC of the proposed system is Thermoplastic Polyurethane 95A (TPU 95A) and the EC is made of Thermoplastic Elastomer 75A (TPE 75A). To calculate the elastic moduli (E) of the materials, uniaxial tension tests were conducted according to ASTM D638. Type IV dog bones were fabricated using the materials for this purpose. The properties of these materials are given below in Table S5.

***Table S5:*** *Material Properties of TPU 95A and TPE 75A*

| **Material Property** | **TPU 95A** | **TPE 75A** |
| --- | --- | --- |
| **Elastic Modulus (E)** | 61.88 MPa  C_01_ = 20.57 MPa  C_10_ = -10.25 MPa | 37.62 MPa  C_01_ = 11.69 MPa  C_10_ = -5.42 MPa |
| **Density** | 1.24 g/cm^3^ | 1.15 g/cm^3^ |

The E values were calculated using the Mooney Rivlin parameters, where E = 6 (C_01_ + C_10_).

In addition to these materials, 3 other materials were used to fabricate the mechanical component of the proposed system to demonstrate experimental force-displacement behaviors as discussed in Section S2.2. Among them, one of them was Polylactic Acid (PLA), another one with carbon fiber reinforced polymer; Polyamide 12 – Carbon Fiber (PA12-CF). The metal part was fabricated using the aluminum alloy – AlSi10Mg.

The silver ink used in the proposed system is the commercially available Metalon HPS-FG32 silver ink which is made of 1.5-micron silver flakes dissolved in butyl-carbitol by 75% in weight. This ink is specifically designed for use on substrates such as plastics.

***S6.2 Experimental Methods***

All the samples were fabricated using additive manufacturing methods. The polymer samples were fabricated using fused filament fabrication technology. For the TPU, TPE and PLA samples, a dual extruder printer - Raise3D Pro3 printer was used. The Raise3D E2CF printer was used to fabricate the samples from PA12-CF. The overhanging elements were printed with support using Poly-vinyl Alcohol (PVA), which were later removed by dissolving in water. All samples were fabricated using a 100% infill and a layer height of 0.2 mm. Laser powder bed fusion was used to fabricate the metal sample. The One Click Metal MPrint printer was used, and the samples were fabricated with a layer height of 20 µm. The silver ink layers were coated using screen printing with a 16T mesh. The loading experiments were conducted using the ADMET 8610 testing machine equipped with a 5 kN load cell. The voltage signals generated from the proposed system were measured using a National Instrument 9220 DAQ module. The current was measured using the Keithley 6514 electrometer.

**References**

[1] *COMSOL Multiphysics*. COMSOL. [Online]. Available: https://www.comsol.com

[2] D. P. Searson, “GPTIPS 2: An Open-Source Software Platform for Symbolic Data Mining,” in *Handbook of Genetic Programming Applications*, A. H. Gandomi, A. H. Alavi, and C. Ryan, Eds., Cham: Springer International Publishing, 2015, pp. 551–573. doi: 10.1007/978-3-319-20883-1_22.

[3] N. Metropolis and S. Ulam, “The Monte Carlo Method,” *J. Am. Stat. Assoc.*, vol. 44, no. 247, pp. 335–341, Sept. 1949, doi: 10.1080/01621459.1949.10483310.
